# Emerging roles of tRNA-derived fragments in cancer

**DOI:** 10.1186/s12943-023-01739-5

**Published:** 2023-02-13

**Authors:** Min Fu, Jianmei Gu, Maoye Wang, Jiahui Zhang, Yanke Chen, Pengcheng Jiang, Taofeng Zhu, Xu Zhang

**Affiliations:** 1 Institute of Digestive Diseases, The Affiliated People’s Hospital of Jiangsu University, Zhenjiang, 212002 Jiangsu China; 2 Jiangsu Key Laboratory of Medical Science and Laboratory Medicine, School of Medicine, Jiangsu University, Zhenjiang, 212013 Jiangsu China; 3 Departmemt of Clinical Laboratory Medicine, Nantong Tumor Hospital/Affiliated Tumor Hospital of Nantong University, Nantong, 226361 Jiangsu China; 4 Department of Pulmonary and Critical Care Medicine, Yixing Hospital Affiliated to Jiangsu University, Yixing, 214200 Jiangsu China

**Keywords:** tRNA-derived fragments, tRNA halves, Cancer, Biomarker

## Abstract

tRNA-derived fragments (tRFs) are an emerging category of small non-coding RNAs that are generated from cleavage of mature tRNAs or tRNA precursors. The advance in high-throughput sequencing has contributed to the identification of increasing number of tRFs with critical functions in distinct physiological and pathophysiological processes. tRFs can regulate cell viability, differentiation, and homeostasis through multiple mechanisms and are thus considered as critical regulators of human diseases including cancer. In addition, increasing evidence suggest the extracellular tRFs may be utilized as promising diagnostic and prognostic biomarkers for cancer liquid biopsy. In this review, we focus on the biogenesis, classification and modification of tRFs, and summarize the multifaceted functions of tRFs with an emphasis on the current research status and perspectives of tRFs in cancer.

## Introduction

Transfer RNAs (tRNAs) are among the most abundant non-coding RNAs (ncRNAs), constituting 4–10% of all cellular RNAs [1]. tRNAs play crucial roles in protein translation by recognizing mRNA codons and transferring specific amino acids to ribosomes for polypeptide chain elongation. Beyond their canonical role in translation, tRNAs also serve as regulators during multiple cellular processes, such as modulating pro-metastatic gene expression [2], regulating neuronal homeostasis with specific isodecoders [3] and mobilizing adaptive translation by base modifications [4, 5]. In 2009, Lee et al. first reported a novel class of small RNAs with second abundance only to miRNAs, termed as tRNA-derived fragments (tRFs) [6]. These fragments are products of tRNA precursors or mature tRNAs cleaved by different ribonucleases at specific positions [6].With the rapid advancement of high-throughput RNA sequencing, increasing amounts of these small RNAs have been discovered [7, 8]. Although tRFs have long been neglected as futile degradation intermediate or by-products of random cleavage, accumulating evidence confirm that tRFs are functional and are associated with diverse human diseases, including cancer [9–12]. This review will summarize the most recent discoveries of tRF biogenesis, classification, modification and biological functions. Furthermore, we highlight the emerging roles of tRFs as biomarkers and therapeutic targets in cancer, hoping to provide novel insights into strategies for cancer diagnosis and treatment.

## Biogenesis and classification of tRFs

**Fig. 1:**
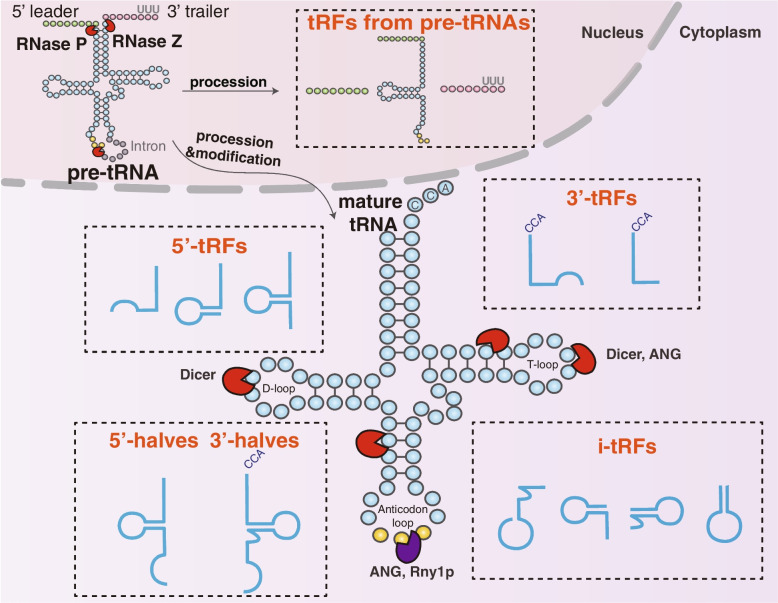
Biogenesis and classification of tRFs. Pre-tRNAs are first transcribed from tRNA genes by RNA polymerase III in the nucleus, followed by cleavage of RNase P and RNase Z at the 5’ and 3’ ends, respectively. The pre-tRNAs undergo splicing of intron by tRNA endonucleases, attachment of CCA sequence at 3’ end and further modifications to achieve tRNA maturation. Based on the incision site of mature tRNAs, tRFs can be divided into 5’-halves, 3’-halves, 5’-tRFs, 3’-tRFs, and i-tRFs. The cleavage of pre-tRNAs also generates tRFs

In 1958, Zamecnik and Hoagland found transfer RNA that could transfer ^14^C-labeled amino acids to synthesized proteins [13]. In the nucleus, the precursor tRNAs (pre-tRNAs) are first transcribed from tRNA genes by RNA polymerase III followed by ribonuclease P and ribonuclease Z cleavage of the 5’ leader and 3’ tail sequences [14, 15]. The pre-tRNAs undergo splicing of intron by tRNA endonucleases, attachment of a CCA sequence at the 3 termini and further modifications during tRNA maturation [16–18]. Conventional tRNAs are 75–93 nt long and highly conserved with four arms (stem and loop): the D-arm, anticodon arm, TψC arm, acceptor arm, and variable arm [19, 20]. tRNAs can be cleaved by different ribonucleases at specific positions, thus generating various tRFs [6]. The biogenesis procedure and classification of tRFs are illustrated in Fig. 1. As classified by MINTbase[17], mature tRNAs can generate 5 subtypes of tRFs: 5’-half, 3’-half, 5’-tRF, 3’-tRF and i-tRF [21].

### 5’-halves and 3’-halves

tRNA halves are generated by the cleavage at the anti-codon site of mature tRNAs. Depending on the relative side of their body against the anti-codon cleavage site, tRNA halves are divided into two subgroups: i) 5’-half, 30–35 nt in length ranging from the 5’ end to the anticodon loop, and ii) 3’-half, 40–50 nt in length ranging from the anticodon loop to the 3’ end [22]. Intriguingly, tRNA halves are often produced asymmetrically, resulting in a much higher abundance of 5’-halves than 3’-halves [23, 24]. Under various stress conditions such as heat shock, UV irradiation, hypoxia, arsenite, amino acid starvation, and viral infection, a specific subtype of tRNA halves is usually produced, termed as tRNA-derived stress-inducible RNAs (tiRNAs) [12, 25, 26]. The distinction between tRNA halves and another tiRNA (transcription initiation RNA) should be noticed [27]. The generation of tRNA halves relies on the catalysis of particular endonucleases including angiogenin (ANG) in mammals or Rny1p in yeast [23, 28]. ANG belongs to the RNase A nuclease family, whose cleavage leaves a cyclic phosphate at the 3’ end of 5’-half and hydroxyl at the 5’ end of 3’-half [23]. Therefore, conventional sequencing protocols are unable to analyze ANG-induced 5’-halves. Treatment with T4 polynucleotide kinase (PNK) can convert the cyclic phosphates to 3’-OH and add a 5’-terminal phosphate, thus facilitating adapter ligation for effective RNA sequencing [29]. Interestingly, a recent study revealed that ANG-knockout only altered the expression of two tRNA halves (the 5’-half from tRNA^HisGTG^ and the 3’-half from tRNA^AspGTC^), with the majority of stress-induced tRNA halves remaining unchanged [30]. This finding suggests that ANG only contributes to partial production of tRNA halves, indicating the existence of other endonucleases involved.

tRNA halves are reported to be mainly located in the cytoplasm [31, 32]. Under stress conditions, ANG travels from the nucleus and accumulates in the cytoplasm, cutting mature tRNAs to produce tRNA halves. Increasing evidence reveal that tRNA halves can also be produced in stress-independent manners. Honda and coworkers found sex hormones and their receptors could induce angiogenin-mediated cleavage of the anticodon loop from aminoacylated mature tRNAs, forming a novel type of tRF, termed as sex hormone-dependent tRNA-derived RNAs (SHOT-RNAs) [33]. The difference between SHOT-RNAs and canonical tRNA halves lies in the 2’, 3’-cyclicphosphate instead of a hydroxyl group at the 3’end of 5’-halves, and an amino acid attached to the 3’ end of the 3’- halves [33]. Another tRNA half named tRNA^Pro^ 5’ half does not show stress-dependent expression either, suggesting the presence of alternative biogenesis pathways [34].

### 5’-tRFs and 3’-tRFs

5’-tRFs are generated by cleavage on the D-loop, D stem or 5’ half of the anticodon stem of tRNAs [35]. The role of Dicer in generating 5’-tRFs is debatable. Cole and coworkers provided the evidence of Dicer involvement in processing 5’-tRFs derived from tRNA^Gln^ by observing a significant decrease of these tRFs after Dicer knockdown [36]. Since Dicer canonically corresponds to double-stranded structures, in some cases the cloverleaf tRNAs may refold into stem-loop hairpins to serve as Dicer substrate with unknown mechanisms [37]. On the contrary, a more recent study showed that Dicer is dispensable for the biogenesis of most 5’-tRFs [38]. The difference in incision loci leads to three subtypes of 5’-tRFs with varying length: i) type a (14–16 nt), ii) type b (22–24 nt), and iii) type c (28–30 nt), with the cleavage sites for type a, b, and c located in the D loop, D stem, and the 5’ half of the anticodon stem, respectively [35]. 3’-tRFs can be produced in Dicer-dependent or Dicer-dependent manners. Dicer, angiogenin, or other RNases cleave on the TψC loop to generate two subtypes of 3’-tRFs: i) type a (~ 18 nt) and ii) type b (~ 22nt), with the length ranging from 13 to 22 nt [35]. The 5’-tRFs mainly exist in the nucleus, while 3’-tRFs mostly reside in the cytoplasm [35, 39].

### i-tRFs

i-tRFs originate from the internal zone of mature tRNAs but does not extend to their 5’ end and 3’ end [40]. Different cleavage starting sites corresponding to different i-tRF subtypes: i-tRFs cleaved at the beginning of the anti-codon loop and overlap with the anticodon loop, i-tRFs formed by cutting the D-stem and contain the D-loop, i-tRFs generated by cleavage at the variable loop and overlap with the variable loop, i-tRFs with an anticodon loop and a stem structure, and etc. [41, 42]. Telonis et al. tended to divide i-tRFs into six subtypes based on the location of the 5’terminus at region A to D, A loop or D loop [43]. Certain i-tRFs derived from tRNA^Glu^, tRNA^Asp^, tRNA^Gly^, and tRNA^Tyr^ are induced during hypoxic conditions in breast cancer cells [42]. The ribonucleases involved in generating i-tRFs remain unknown.

### tRFs from pre-tRNAs

Apart from the standard classification of tRFs by MINTbase [21], there are atypical classes of tRFs generated from pre-tRNAs. tRF-1 s are derived from the 3’ trailer fragment of precursor tRNAs trimmed by RNaseZ or its cytoplasmic homologous endonuclease ElaC ribonuclease Z 2 (ELAC2) [44]. tRF-1 s 16–48 nt in length start after the 3’ end of the mature tRNA before CCA addition and end with a ‘poly-U’ sequence (UUUUU, AUCUU, UUCUU, or GUCUU) [45]. Though originated from pre-tRNAs, tRF-1 s are mostly cytoplasmic [44]. Therefore, the tRF-1 s are believed to be translocated to the cytoplasm after generation in the nucleus [46]. However, it is also reported that tRF-1 s are produced and regulated by ELAC2 directly in the cytoplasm, e.g., tRF-1001 [6]. 5’U-tRFs, mostly 17 nt in length, are processed from the pre-tRNA 5’ leader sequences of various tRNAs [41]. They were identified by mapping the sequencing reads of RNA from human prostate cancer tissues to a tRNA reference database [41]. The abundance of tRF-1 and 5’U-tRF is generally lower than 5’-tRF and 3’-tRF [6, 41]. The pre-tRNAs also generate another type of tRF, named 5’ leader-exon tRF [47]. These 5’ leader-exon tRFs start from the very beginning of the 5’ leader sequence and contain the 5’ exon sequence of the pre-tRNAs [47]. Inactivation of RNase CLP1 kinase activity leads to the poor generation of tRNA exon halves but the accumulation of 5’ leader-exon tRF in motor neurons, possibly caused by the defect in tRNA exon ligation in a CLP1 kinase-dead background driven by oxidative stress [47].

## tRNA modification in tRF biogenesis

The underlying mechanisms regulating tRFs biogenesis remain unclear. However, emerging evidence prove that the modifications of tRNAs not only influence the stability and function of tRNAs but also regulate tRF biogenesis.

### Methylation

Methylation is the most prominent post-transcriptional modification of tRNAs. The DNA methyltransferase 2 (DNMT2) methylates C5 at cytosine residues (m5C) of specific tRNAs at cytosine 38 (C38) [48]. Deletion of Dnmt2 could induce the loss of m5C at the C38 position, leading to enhanced stability of tRFs against RNase degradation [49]. Similarly, NOP2/Sun RNA methyltransferase 2 (NSUN2) and tRNA methyltransferase 2 homolog A (TRMT2A) performs post-transcriptional methylation of tRNAs at m5C and 5-methyluridine (m5U) respectively [24, 50]. Deletion of NSUN2 and TRMT2A induces angiogenin overexpression and accumulation of tiRNAs [24, 50]. Besides, bicoid interacting 3 domain containing RNA methyltransferase (BCDIN3D) mediates the phospho-methylation of tRNA^His^, resulting in increased resistance against Dice and regulation of 3’-tRF formation [51]. On the other hand, demethylases ALKBH1 and ALKBH3 can remove the methyl group of m1A in tRNAs, which harbor increased sensitivity to the cleavage of angiogenin to produce tRFs [52, 53].

### Pseudouridylation

Pseudouridylation (Ψ) is driven by evolutionarily conserved pseudouridine synthases (PUSs), with psuedouridine converted from uridine by pseudouridine synthetase (PUS7) [54]. In human embryonic stem cells (hESCs), PUS7 binds to distinct tRNAs and controls the biogenesis of certain tRFs [54]. The deletion of PUS7 results in Ψ loss of tRNAs, leading to the decreased expression of certain 5’-tRFs derived from tRNAs containing a 5’ terminal oligoguanine (TOG), such as tRNA-Ala, tRNA-Cys, and tRNA-Val [54].

### Queuosine modification

Queuosine (Q) is a hypermodified 7-deaza-guanosine which substitute guanine at the wobble position 34 of tRNA^His^, tRNA^Asn^, tRNA^Tyr^, and tRNA^Asp^ with 5’GUN anticodons [55]. In human, Q modification is accomplished by the incorporation of queuine into the wobble anticodon nucleotide by the heterodimeric enzyme of QTRT1/QTRT2. Q modification can directly protect cognate tRNA^His^ and tRNA^Asn^ from angiogenin cleavage in HEK293T cells [56]. Intriguingly, Q modification may directly affect the Dnmt2-dependent m^5^C38 modification in tRNA^Asp^ of fission yeast, indicating the crosstalk between different modification types [57, 58].

### 2'-O-methylation

Methylation of ribose at 2’-OH group can occur in all four nucleotides and other non-canonical nucleotides. Therefore, 2’-O-methylation in cellular RNAs is widely spread in all domains of life. In bacteria, the tRNA halves originated from tRNA^Asp^ and tRNA^Arg^ by ribotoxins can be further repaired by Pnkp/Hen1 heterotetramer to produce full-length tRNAs with 2’-O-methylation at the cleavage site, which enhances tRNA resistance against ribotoxins [59]. Furthermore, 2’-O-methylation of the wobble cytidine at C34 in HAP1 cells protects human elongator tRNA^Met^ (CAT) from endonucleolytic cleavage by angiogenin [60]. 2’-O-methylation limits the endonucleolytic cleavage of tRNA^Phe^ by TRM7/FTSJ1 and stabilizes tRNA^Phe^ fragments in *Drosophila* [61]. Besides, bacterial tRNA 2’-O-methylation is recently found dynamically regulated under stress conditions [62], further indicating the association between this type of modification and stress-induced tRF biogenesis.

Apart from the modifications mentioned above, more types of modifications involved in tRFs needs to be unveiled. The mechanism of these modifications affecting tRF biogenesis is not well characterized. One possible explanation is that the structures of modified tRNAs are too tight or stable for nucleases to access. However, whether this procedure is totally the consequence of RNA folding or there are proteins participate in needs further investigation.

## tRF function

**Fig. 2:**
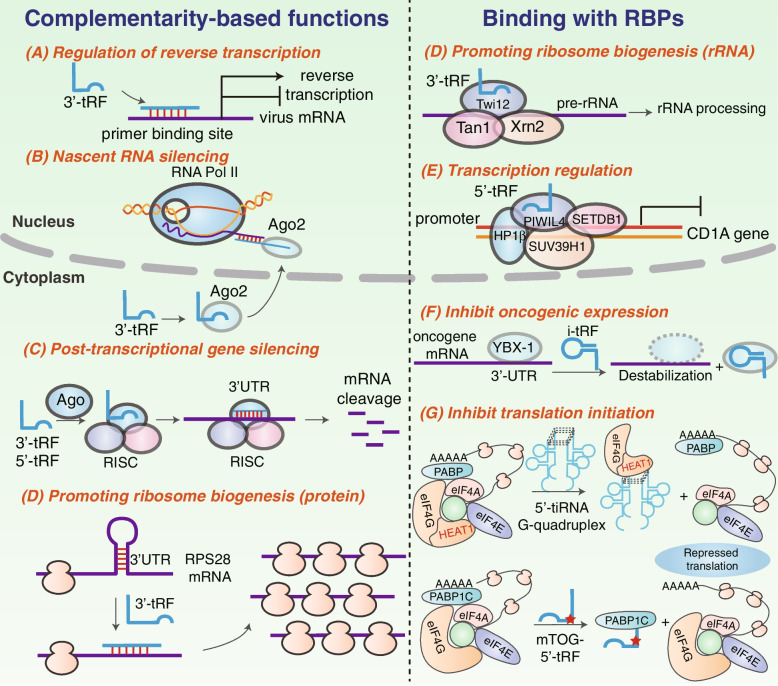
Primary functions of tRFs, based on complementarity or binding with RBPs. **A** Regulation of reverse transcription, **B** Nascent RNA silencing, **C** Post-transcriptional gene silencing, **D** Promoting ribosome biogenesis, **E** Transcription regulation, **F** Inhibit oncogenic expression and **G** Inhibit translation initiation

Although the investigation of tRF biology is still in its infancy, emerging evidence has revealed the diverse functional roles of tRFs. The distinct functionalities of tRFs are illustrated in Fig. 2.

### Nascent RNA silencing

The mechanism of nuclear RNA interference is controversial, due to the skepticism over the presence of nuclear RNAi factors and technical difficulties [63]. Very recently, a novel nascent RNA silencing mechanism of Dicer-dependent tRFs has been reported [9]. The Dicer-generated tRFs associate with AGO2 proteins and target introns of nascent RNA through base pairing, followed by AGO2 slicing and the translational prevention. Unlike post-transcriptional/transcriptional gene silencing, nascent RNA silencing takes place in nuclei and has no effect on the transcriptional level of target genes. Of note, the single-stranded tRFs directly bind to nuclear AGO2, coherent with the previously reported absence of duplex RNA in nuclear RNAi progress, probably due to the lack of RISC loading and maturation factors in the nucleus [64]. However, the entrance of tRF to RISC regardless of its single-strand characteristic remains enigmatic.

### Transcriptional regulation

Pekarsky et al. performed an RNA immunoprecipitation and revealed that ts-53 and ts-101 were detected in complexes containing AGO1 and AGO2 and in complexes containing Piwi-L2, indicating that tRFs are capable of associating with both AGO and Piwi proteins [65]. However, the evidence is inadequate for the definition of these fragments as functional PIWI-interacting RNAs (piRNAs), as proposed by Genzor et al. [66]. Zhang and coworkers discovered a 5’-tRF, tRNA-Glu-derived piRNA (td-piR (Glu)), which could bind to PIWIL4 and recruit SETDB1, SUV39H1, and heterochromatin protein 1β (HP1β) to the CD1A promoter region, thus facilitating H3K9 methylation and significantly inhibiting CD1A transcription in human monocytes [67]. Besides, Chen et al*.* suggested that sperm tRFs preferentially bind to promoter regions rather than coding regions to facilitate regulation of downstream genes, resulting in altered metabolic trait inheritance and embryonic development [68].

### Post-transcriptional gene silencing

Since tRFs share similar length distribution with miRNAs and bear satisfactory sequence complementarity for mRNAs, the preliminary studies mainly focus on their miRNA-like roles in post-transcriptional gene silencing. The meta-analysis of human photoactivatable-ribonucleoside-enhanced crosslinking and immunoprecipitation (PAR-CLIP) data from Kumar et al. showed a prominent preference for 5’-tRFs and 3’-tRFs to associate with AGO1, 3, and 4 rather than AGO2, while there is almost no incorporation for tRF-1 s with all four AGOs [35]. The comparable read counts and positional T to C mutational frequency of 5’-tRFs and 3’-tRFs indicate that these tRFs associate with AGOs in a manner similar to miRNAs to regulate gene silencing [35]. The preference for tRFs with certain AGOs was later found to be modulated in an age-dependent manner in *Drosophila* [69], indicating a dynamic regulation of tRFs from young to adult organisms. Either Dicer-dependent or -independent tRFs are able to associate with AGOs to generate RNA-induced silencing complex (RISC), which is further guided to the partially complementary site of target mRNAs (mostly 3’UTR region) and leads to translational repression as well as mRNA decay [70, 71]. Apart from 5’-tRF, 3’-tRF is also found associated with Twi12 protein, a PIWI protein essential for growth, to activate exonuclease Xrn2 and Tan1 for rRNA processing in *Tetrahymena* [72]*.* In another case, ts-53 and ts-101 interact with PiwiL2 protein as well as AGO protein and are downregulated in cancer [73], indicating their potential regulatory roles in gene silencing.

Furthermore, tRFs may regulate post-transcriptional gene expression by directly binding to RNA-binding proteins (RBPs). Y box binding protein 1 (YBX1 or YB-1) is a versatile RNA-binding protein involved in multiple cellular pathways and linked with cancer progression [74, 75]. A group of i-tRFs derived from tRNA^Glu^, tRNA^Asp^, tRNA^Gly^, and tRNA^Tyr^ compete with YBX1 to bind to the 3’UTRs of multiple oncogenic transcripts in breast cancer cells, thus eliminating the stabilization effect of YBX1 [42]. The mRNA destabilization results in decreased expression of these oncogenes, ultimately inhibiting cancer metastasis [42]. In another scenario, tsGlnCTG showed interaction with IGF2BP1, an RNA binding protein that stabilizes c-Myc mRNA, resulting in inhibited transcript stability and promoted differentiation of mouse embryonic stem cells [76].

### Translational regulation—inhibition of translation initiation

Increasing evidence suggest that tRFs can modulate global translation process, acting as both negative and positive regulators. As mentioned above, ANG induces the formation of tRNA halves under stress conditions. Of note, specific ANG-induced 5’-tiRNAs (but not 3’-tiRNAs) such as 5’-tiRNA^Ala^ and 5’-tiRNA^Cys^ inhibit translation initiation by cooperating with the translational repressor YB-1 to displace the cap-binding complex eIF4G/A/F from capped mRNA and inducing the assembly of stress granules (SGs) [77]. Although required for packaging tiRNA-repressed mRNAs into SGs, YB-1 is dispensable for tiRNA-mediated translational repression [78]. It was further confirmed that the activity of these selected 5’-tiRNAs relies on the formation of a G-quadruplex (G4) structure assembled from four 5’-tiRNA molecules with the interaction of theTOG motifs (four to five guanine residues) at their 5'-ends [79, 80]. Subsequent work uncovered that the G4-tiRNAs are capable of directly binding to the HEAT1 domain of eIF4G, leading to the impairment of 40S ribosome scanning and the translation shutdown [81].

Apart from tRNA halves, 5’-tRFs can also regulate translational initiation by aberrant modifications. Bellodi’s group proposed that the Ψ modification at U8 mediated by PUS7 in embryonic stem cells can drive the resulting mini TOG (mTOG)-containing 5’-tRFs to bind to polyadenylate-binding protein 1 (PABPC1), an integral protein responsible for eIF4G/A interaction and initiation of cap-dependent translation. The formation of mTOG-PABPC1 complex blocks PABPC1 recruitment to eIF4F, thus repressing protein translation. In contrast, PUS7 loss impairs this tRF-mediated translational regulation in embryonic stem cells, resulting in increased protein biosynthesis and early defective germ layer specification [54]. Another study uncovered that the absence of the cytosine-5 RNA methyltransferase NSun2 causes the accumulation of 5’-tRFs and reduction of protein translation rates [24]. However, how the failure in NSun2-mediated tRNA methylation induces translation arrest remain elusive. As indicated by the above findings, specific modifications could regulate not only tRF biogenesis but also post-transcriptional gene expression which directly impacts their biological functions.

### Translational regulation—promotion of protein translation by regulating ribosome biogenesis

tRFs also regulate translation by accelerating ribosome biogenesis. A specific 3’-tRF derived from the Leu-CAG tRNA (LeuCAG3’tsRNA) interacts with two ribosomal protein mRNAs (RPS28 and RPS15) to enhance their translation and increase tumor cell viability [82]. Mechanically, the LeuCAG3’tsRNA maintains the translation of RPS28 and ultimately the number of ribosomes by binding to the 3’-UTR and coding regions in RPS28 mRNA, leading to the unfolding of its secondary structure and enhanced translation [83]. The knockdown of LeuCAG3’tsRNA induces substantial apoptosis in HeLa cells and patient-derived orthotopic hepatocellular carcinoma (HCC) xenografts in mice models, indicating the contribution of 3’-tRF in cancer progression [82]. The same group further demonstarte that the LeuCAG3’tsRNA is fully aminoacylated and its generation is highly regulated by the leucyl-tRNA synthetase (LARS1), which conjugates the specific amino acids with cognate tRNAs [84]. Another study suggests that 19-nucleotide 5’-tRF Gln19 specifically interacts with the ternary multisynthetase complex (MSC) and promotes ribosomal and poly(A)-binding translation elongation in HeLa cells [85]. Moreover, the conserved GG-dinucleotide motif at the 3’ends in 5’-tRFs is essential for the interaction with MSC, leading to the destabilization of MSC and the inhibition of ribosome maturation [85, 86].

### Inhibition of apoptosis

Apart from the effects on gene silencing and protein translation, tRFs also regulate cell apoptosis. In wild-type mouse embryonic fibroblasts (MEF), hyperosmotic stress induces apoptosis following the release of cytochrome c (Cyt c) from mitochondria and subsequent formation of apoptosomes [87]. Saikia et al. propose that ANG treatment protects MEFs or primary neurons from hypertonicity-induced apoptosis by inducing the generation of 5’- and 3’-tiRNAs to sequester cytosolic Cyt c within a ribonucleoprotein complex [88]. The restraint of Cyt c leads to the decreased formation of apoptosome or inhibited apoptosome activity, ultimately protecting cells from apoptosis [88].

### Regulation of reverse transcription

Transposable elements (TEs) are mobile genomic DNAs which induce autonomous or non-autonomous transposition, leading to potentially harmful heterochromatins [89]. Therefore, TE transcription is tightly controlled by epigenetic marks such as DNA methylation and histone modifications. However, alternative regulatory networks are indicated when these epigenetic marks are absent. Recently, tRFs have been confirmed to protect the host genome from TE intervention [71, 90]. The 18 nt 3’-tRFs specially inhibit mouse LTR-retrotransposon (also known as endogenous retrovirus (ERV)) activity by competing with intact tRNAs for the highly conserved primer binding site (PBS) of LTR-retrotransposon, leading to the blockade of the ERV reverse transcription [90]. Meanwhile, the 22 nt 3’-tRFs affect transposon expression by post-transcriptionally silencing of coding-competent ERV mRNA by binding to the PBS sequence [90]. In general, the two 3’-tRFs inhibit LTR-retrotransposon activity by varied mechanisms but both depending on base complementarity with PBS sequence. Similarly, an 18 nt 3’-tRF derived from the dsRNA hybrid formed by the HIV-1 PBS and the 3’ termini of the human cellular tRNAlys3 can in turn inhibit HIV-1 reverse transcription by binding to AGO2 and targeting PBS, resulting in suppression of HIV-1 [91]. In another study, tRF-3019 has been reported to act as a primer for human T cell leukemia virus type 1 (HTLV-1) reverse transcriptase by binding to PBS, thus enhancing viral infection [92]. These findings demonstrate that tRFs can play dual roles in regulating viral reverse transcription and potentially serve as novel targets for viral control.

### Regulation of noncoding RNA production and chromatin accessibility

In addition, the recent studies indicate that tRFs can modulate noncoding RNA biogenesis and global chromatin organization [93, 94]. A 28 nt 5’-tRF called 5' tRF-Gly-GCC, or tRF-GG for brevity was identified as an inhibitor of genes associated with the endogenous retroelement MERVL [93]. Interestingly, the repression of MERVL-associated genes is found to be a downstream result of an evolutionarily conserved function for tRF-GG in promoting noncoding RNA production [94]. The tRF-GG interacts with hnRNPF/H and positively regulates the production of noncoding RNAs in Cajal bodies, including the U7 snRNA, which modulates heterochromatin-mediated transcriptional repression of MERVL elements by supporting an adequate supply of histone proteins [94]. In this way, tRF-GG manipulation alters a variety of noncoding RNA production and chromatin accessibility of specific genes in both mouse ES cells and preimplantation embryos [94].

## Biological roles and clinical values of tRFs in cancer

In 2018, Pliatsika et al*.* released the MINTbase v2.0 which contains the largest collection of 26,531 distinct human tRFs by analyzing all The Cancer Genome Atlas (TCGA) datasets as of October 2017 [95]. This database has provided the researchers a useful tool for in-depth investigation of tRFs in cancers. Previously, the same group first analyzed breast cancer datasets from TCGA and found that the identity and abundance of tRFs exhibit race-dependent differences [40]. Later, the correlation networks constructed by them uncovered race/ethnicity-dependent tRF-mRNA associations in prostate adenocarcinoma and triple-negative breast cancer [96, 97]. More recently, this work has been extended to a large-scale pan-cancer analysis to investigate the tRF-mRNA associations in 32 cancer types of TCGA cohort [43]. Some major points have been made for investigating the commonalities of tRFs in cancer. First, tRF generation strongly depends on tRNA genomic origin in terms of tRF identity and abundance [43]. Second, both nuclear and mitochondrial tRFs are positively correlated with shorter genes with high density of repeats, and negatively correlated with longer genes with low density of repeats in most of the 32 cancer types [43]. Third, tRF-mRNA correlations are cancer-specific while uncovering several pathways universally regulated by tRFs among multiple cancer types [43]. Fourth, in addition to acting like miRNAs or decoying RBPs, tRFs could interact with ribosomal proteins and aminoacyl-tRNA synthases in many cancer types [43]. Finally, some pathways identified with tRF association in bladder, lung, and kidney cancers are regulated in a sex-dependent manner [43]. This large-scale analysis supports the general and extensive role of tRFs in posttranscriptional regulation in cancer.

On the other hand, the aberrant expression and function of specific tRFs have also been reported in various cancers, including breast cancer (BC), gastric cancer (GC), and colorectal cancer (CRC), among others (Table 1). Growing evidence demonstrate that the dysregulation of tRFs is closely related with clinicopathologic features and survival of cancer patients [98–100]. Functional studies indicate that tRFs are involved in various biological activities during cancer development and progression, such as tumor cell proliferation, metastasis, apoptosis and chemoresistance [101–103]. Herein, we will summarize the most recent advances of the biological roles and clinical values of tRFs in different cancers, as well as the associated mechanisms and regulatory networks.

**Table 1:** The deregulated tRFs in cancer

Cancer type	Expression level	tRF	Sample type	Clinical significance	Ref
Breast cancer	Upregulated	SHOT-RNAs	Tissue	SHOT-RNAs are more enriched in ER^+^ BC tissues than triple-negative BC and normal breast tissues	[33]
		tRF-19-W4PU732S	Tissue	High level expression predicts poor survival	[104]
		tRF-34-RKVP4P9L5FZUHM	Tissue	High expression correlates with poor survival of BC patients	[105]
		nlr-18-949M9Y0Q, nlr-20-D6PUMZQZ	Tissue	Higher levels of both tRFs predict poorer OS; In the T cell exhaustion group, low level of nlr-18-949M9Y0Q predicts better OS	[106]
		tRF-32-XSXMSL73VL4YK	Tissue	No obvious correlation	[107]
		nlr-31-W01KN44VD3YZE	Tissue	Not mentioned	[108]
		nlr-30-W01KN44VD3YZ, nlr-32-FKSZZ6KSNNQ7Q, tRF-34-PUDP4PZKFJ7PEP	Plasma	High expression of nlr-30-W01KN44VD3YZ and tRF-34-PUDP4PZKFJ7PEP predict worse DFS and OS	[98]
		tRF-32-PS5P4PW3FJHP1	Serum, Ev from serum	High diagnostic accuracy for BC is obtained in a model with miR-21-5p (3’ addition C) and miR-23a-3p	[109]
	Downregulated	tRF-17-SMKVNM1, tRF-20-6NMH490V, and tRF-20-VIPZK0JN	Tissue	tRF levels decrease in metastatic samples compared to primary tumors	[42]
		nlr-14-7O58FX, nlr-21-F0L9XZ30E, nlr-34-5QKDN6QQ1362HQ	Tissue	Higher level of each tRF predicts improved OS; In the T cell exhaustion group, high level of nlr-34-5QKDN6QQ1362HQ predicts improved survival	[106]
		tRF-32-Q99P9P9NH57SJ, tRF-17-79MP9PP	Tissue, serum	Lower expression correlates with higher stage progression and lymph node metastasis	[107, 110, 111]
		tRF-18-YRRHQFD2, tRF-18-HSQSD2D2, tRF-20-B2NZW7O6	Tissue, serum	Expression levels are also downregulated in early-stage BC patients	[112]
		tDR-000620	Serum	Low tDR-000620 expression is an independent adverse predictive factor for recurrence-free survival of triple-negative BC	[113]
		nlr-21-F5W8E7OME, tRF-18-18VBY9DV, tRF-23-NB57BK87DZ	Serum	Expression levels are downregulated in non-triple-negative BC	[114]
		tRF-32-1HPSR9O9337KF	Plasma	tRF-32-1HPSR9O9337KF levels significantly decrease in the blood of patients with HER2^+^ BC reflecting tumor status (control > early cancer > metastatic cancer)	[115]
		tRF-18-HR0VX6D2, iso-26-QO608YX91ZE	Plasma	They are better biomarker candidates than their corresponding miRNAs	[116]
		tRF-16-87R8WPE, tRF-22-PNR8YP9LL, tRF-23-PNR8YP9LD6, tRF-16-SP5830D, tRF-24-OB1690PQIY, tRF-28-OB1690PQR304	Plasma, exosomes from plasma and tissues	Low expressed tRF-16-87R8WPE of HER2^+^ early BC patients correlates to worse DFS and OS	[8]
Gastric Cancer	Upregulated	tRF-19-FRJ4O1E2	Tissue	Higher tRF-19-FRJ4O1E2 correlates with higher lymph node metastasis	[117]
		tRF-18-8R1546D2	Tissue	tRF-18-8R1546D2 can discriminate GC stage I-II and III-IV, lymph node metastasis and differentiation	[118]
		nlr-18-08P2F5DF	Tissue	Expression is positively correlated with tumor size and the depth of tumor invasion	[119]
		tRF-29-RRJ89O9NF5JP	Tissue, serum	Expression is positively correlated with the degree of lymph node metastasis and tumor grade	[120]
		tRF-23-Q99P9P9NDD	Tissue, serum	Expression is positively associated with T stage, lymph node metastasis, TNM stage, and nerve/vascular invasion	[121]
		tRF-31-U5YKFN8DYDZDD	Serum	Expression is positively associated with depth of invasion, lymph node metastasis, TNM stage, and vascular invasion	[122]
	Downregulated	nlr-15-D47RMZ,nlr-17-XI61Z7Q	Tissue	Lower expression correlates with larger tumor size and poorer differentiation	[123, 124]
		tRF-24-V29K9UV3IU, tRF-34-Q99P9P9NH57S15	Tissue	Not mentioned	[125–127]
		tRF-34-86V8WPMN1E8Y2Q	Tissue, plasma	Lower expression correlates with larger tumor size	[128]
		tRF-18-79MP9P04	Tissue, plasma	Lower expression correlates with larger tumor size, TNM stage, lymph node metastasis and poorer survival	[99]
		tRF-33-P4R8YP9LON4VDP	Plasma	Not mentioned	[101]
		tRF-19-3L7L73JD	Plasma	Higher expression predicts smaller tumor size	[129]
Colorectal cancer	Upregulated	nlr-26-LIKFW7X7JNE	Tissue	nlr-26-LIKFW7X7JNE is upregulated in both colon adenomas and adenocarcinomas	[73]
		tRF-phe-GAA-031, tRF-VAL-TCA-002	Tissue	Increased expression positively correlates with distant metastasis and clinical stage, and predicts poorer survival	[130]
		tRF-32-6978WPRLXN48Q	Tissue	High tRF-32-6978WPRLXN48Q level predicts poor DFS, OS, and high recurrence	[131]
		tRF-35-PW5SVP9N15WV7W	Tissue	Expression correlates positively with tumor size, but not with age, sex, or clinical stage	[12]
		tRF-34-Q99P9P9NH57S15, tRF-30-ROD8N0X0JYOY, tRF-29-R9J89O9NF5JP, tRF-18-8R1546D2	Tissue	Not mentioned	[132, 133]
		tRF-33-79MP9P9NH57SD3	Tissue, serum	Increased expression is positively associated with lymph node and distant metastasis	[134]
		tRF-35-PNR8YP9LON4VN1	Plasma	Increased expression positively correlates with pathological stage and metastasis	[135]
	Upregulated/downregulated	tRF-18-HSRVK7D2, tRF-33-PSQP4PW3FJI0W, tRF-33-PSQP4PW3FJIKW, tRF-32-O7M8LOMLQHWU3, tRF-18-H9Q867D2, tRF-16-I3FJQSD	Tissue	They are included in a six-tRF-based prognostic model with high risk score predicting worse survival, especially for stage III patients	[133]
	Downregulated	tRF-16-79MP9PE, tRF-16-RP9830D, tRF-16-RPM830E, tRF-22-WB86Q3P92, tRF-22-WE8SPOX52, tRF-22-WE8S68L52, nlr-17-863IP5J	Tissue	Not mentioned	[132, 133, 136]
Lung cancer	Upregulated	nlr-15-395P4P	Tissue	High expression is associated with postoperative recurrence and poor prognosis	[137]
		nlr-34-4P5830MMUKLYHE	Tissue, serum	nlr-34-4P5830MMUKLYHE is greatly upregulated in stage III and stage IV cases, and increases with stage progression	[138]
		nlr-28-PNR8YP9LONDQ	Tissue, plasma	High nlr-28-PNR8YP9LONDQ predicts poor survival of NSCLC	[139]
		tRF-21-RK9P4P9L0, tRF-16-PSQP4PE	Tissue, serum	tRF-21-RKP4P9L0 is negatively associated with prognosis in lung adenocarcinoma	[140]
		tRF-31-79MP9P9NH57SD	Serum	Expression is positively associated with clinical stage and the malignancy of lymph node	[141]
		tRF-30-RK9P4P9L5HMV, tRF-31-RK9P4P9L5HMVE, tRF-26-MI7O3B1NR8E, tRF-27-WJ9X0UD394N, tRF-26-SP5830MMUKD, tRF-29-MIF91SS2P4IR, tRF-30-3JVIJMRPFQRD, tRF-31-ROD8N0X0JYOYE, tRF-32-ROD8N0X0JYOYO	Peripheral blood mononuclear cells	TRY-RNA signature precisely discriminates between control, lung cancer, and pulmonary tuberculosis subjects	[142]
	Downregulated	nlr-29-P9MWYRKHNZI2, iso-25-QO608YX91Z, nlr-33-Z4ODD8U82KP90Q, nlr-31-SU12RXXNBZY3E	Tissue	Not mentioned	[65, 73]
		tRF-16-L85J3KE	Tissue, serum	No significant correlation with prognosis is observed	[140]
		AS-tDR-007872	Tissue, plasma	AS-tDR-007872 is more sensitive in distinguishing stage III and IV than stage I-II NSCLC patients	[143]
Leukemia	Upregulated	tRF-21-ZPEK45H5D	Peripheral blood mononuclear cells	Overexpression of tRF-21-ZPEK45H5D is related to poor OS of the CLL patients	[144]
	Downregulated	i-tRF of tRNA^GlyCCC^	Peripheral blood mononuclear cells	Reduced OS for CLL patients with positive expression of this i-tRF	[145]
		nlr-29-P9MWYRKHNZI2, iso-25-QO608YX91Z, nlr-33-Z4ODD8U82KP90Q, nlr-31-SU12RXXNBZY3E	Peripheral blood mononuclear cells	Not mentioned	[65, 73]
		tDR-Asp family	Bone marrow	Decreased tDR-Asp can predict myelodysplastic syndromes that are likely to progress to acute myeloid leukemia	[146]
	NOT significant	tRF-18-5J3KYU05, tRF-18-HR0VX6D2	Peripheral blood mononuclear cells	High expression levels are associated with inferior OS	[147, 148]
Pancreatic cancer	Upregulated	AS-tDR-000064, AS-tDR-000069, AS-tDR-000102	Tissue	Not mentioned	[149]
		tRF-18-HR0VX6D2	Tissue	Not mentioned	[150]
		tRF-30-6978WPRLXN4V, tRF-18-HRERXFD2	Tissue, serum	The tRF-30-6978WPRLXN4V-high and/or tRF-18-HRERXFD2-high PC patients show significantly worse prognosis	[151]
		tRF-41-N5EX62Z6EXEY0VWUD, tRF-37-KSBE78YLKZKWE52, tRF-23-YOY9Q867D2	Tissue, serum	High tRF-41-N5EX62Z6EXEY0VWUD expression is associated with advanced tumor stage and liver metastasis, and predict poor OS	[152]
	Downregulated	AS-tDR-001391	Tissue	Not mentioned	[149]
		nlr-33-OJ5B8LK7F1BUDV	Tissue	Low expression is associated with advanced TNM stage, N stage and predicts poor survival	[153]
		tRF-21-VBY9PYKHD	Tissue	Patients with low expression levels have poor prognosis	[154]
Liver cancer	Upregulated	tRF-30-PNR8YP9LON4V	Tissue	High expression predicts poor survival	[155]
		tRF-40-EFOK8YR951K36D26, tRF-34-QNR8VP94FQFY1Q, tRF-32-79MP9P9NH57SJ, tRF-31-87R8WP9N1EWJ0	Plasma exosomes	Not mentioned	[156]
Ovarian cancer	Upregulated	nlr-24-5D0RKRKNFQ, nlr-25-756BIW7K5Z	Tissue	Not mentioned	[73]
		tRF-32-P4R8YP9LON4V3, tRF-25-9NJ4S2I7L7	Serum	Not mentioned	[157]
		i-tRFs of tRNA^GlyGCC^	Tissue, serum	Increased expression is associated with advanced stages, suboptimal debulking and early progression, and predicts poor OS of early ovarian cancer	[158]
		tRF-03357, tRF-03358	Serum	Expression level is significantly increased in high-grade serous ovarian cancer	[159]
	Downregulated	tRF-28-PNR8YO9LOND5, tRF-28-PNR8YP9LOND5	Serum	Not mentioned	[157]
Prostate cancer	Upregulated	SHOT-RNAs	Tissue	SHOT-RNAs are upregulated in AR^+^ prostate cancer	[33]
Bladder cancer	Upregulated	tRF-34-Q99P9P9NH57S15, tRF-32-P4R8YP9LON4V3, tRF-31-P4R8YP9LON4VD	Tissue	Not mentioned	[160]
		tRF-29-PSQP4PW3FJF4	Tissue, serum	Increased expression is associated with aggressive tumor phenotype, early disease progression and poor treatment outcome	[161]
	Downregulated	tRF- + 1:T20-Ser-TGA-1	Tissue	Not mentioned	[160]
Renal cancer	Downregulated	5’ halves of tRNA^ValAAC^	Tissue	Expression level is inversely correlated with staging and grading	[162]
		5’ halves of tRNA^ArgCCT^, tRNA^GluCTC^,and tRNA^LysTTT^	Tissue, serum	Lower expression levels are associated with adverse clinicopathological parameters	[163]
Esophageal squamous cell cancer	Upregulated	tRNA-GlyGCC-5	Salivary exosomes	Patients with high risk score have shorter OS and progression-free survival, and benefit more from adjuvant therapy	[164]
Multiple myeloma	Upregulated	tRF-34-86J8WPMN1E8Y2Q	Tissue	Not mentioned	[165]
		tRF-18-5J3KYU05, tRF-18-HR0VX6D2	CD138 + plasma cells	The OS and PFS are significantly longer in MM patients with high tRF-18-5J3KYU05 or tRF-18-HR0VX6D2 levels	[166]
	Downregulated	tRF-17-8YQ84V2	Tissue	Not mentioned	[165]
	Unkown	tRF-60:77-Thr-TGT-1,tRF-22-PY5P4PZ3H	Tissue	tRF-60:77-Thr-TGT-1 is upregulated and tRF-22-PY5P4PZ3H is downregulated in relapsed/refractory MM compared with newly diagnosed MM	[103]
Cervical cancer	Upregulated	tRF-22-ZRS3S3RX5	Tissue	Expression is positively associated with the gastric subtype of endocervical adenocarcinoma	[167]
Papillary thyroid cancer	Upregulated	tRF-32-PNR8YP9LON4V3, tRF-39-0VL8K87SIRMM12E2	Tissue	Not mentioned	[168, 169]
Lymphoma	Downregulated	tRF-22-WE8SPOX52	Tissue	Not mentioned	[170]
Pharyngeal cancer	Upregulated	tRF-30-PSQP4PW3FJIK	Tissue	Increased expression closely relates to tumor staging, differentiation grade, smoking history and drinking history, and predicts metastasis	[100]
		tRF-28-Q99P9P9NH50E, tRF-24-QW58309N0Y	Tissue	Not mentioned	[171]
	Downregulated	tRF-22-WD8YQ84V2	Tissue	Not mentioned	[171]
Laryngeal cancer	Downregulated	tRF-33-Q1Q89P9L842205	Tissue	Low expression is closely associated with lymph node metastasis and advanced stages	[172]
Head and neck squamous cell carcinoma	Upregulated	tRF-20-S998LO9D	Tissue	High expression level is significantly associated with poor OS	[173]
	Upregulated in tissue Downregulated in serum	5’ tRNA-Val-CAC-2–1 half	Tissue, serum	Not mentioned	[174]
Uveal melanoma	Downregulated	tRF-22-BP4MJYSZH, tRF-21-45DBNIB9B	Tissue	Low expression relates to various molecular phenotypes, metastatic disease, and poor patient survival	[175]
Glioma	Downregulated	nlr-20-2IHZI73Z, tRF-18-HRE9XFD2, and tRF-22-WB86N7O52	Tissue	High expression correlates with positive IDH mutant and better survival	[176]
Cholangiocarcinoma	Upregulated	tRF-20-LE2WMK81	Tissue	Not mentioned	[177]
	Downregulated	tRF-34-JJ6RRNLIK898HR, tRF-38-0668K87SERM492V, tRF-39-0668K87SERM492E2	Tissue	Not mentioned	[177]

The names of tRFs are converted to the standardized license plate notation as possible [21]

*Abbreviations*: *SHOT-RNAs* Sex HOrmone-dependent TRNA-derived RNAs, *ER* Estrogen receptor, *BC* Breast cancer, *OS* Overall survival, *DFS* Disease-free survival, *EV* Extracellular vesicles, *GC* Gastric cancer, *NSCLC* Non-small cell lung cancer, *CLL* Chronic lymphocytic leukemia, *PC* Pancreatic cancer, *AR* Androgen receptor; *MM* Multiple myeloma

### tRFs in BC

To date, BC is the most studied cancer for tRFs. Based on high-throughput sequencing, the expression profiles of tRFs have been characterized and increasing deregulated tRFs were identified in BC such as tRF-32-XSXMSL73VL4YK, tRF-32-Q99P9P9NH57SJ, tRF-17-79MP9PP, and tsRNA-26576 [107, 108]. Some of these tRFs were further validated as potential diagnostic or prognostic biomarkers for BC. For instance, circulating tRF-Arg-CCT-017, tRF-Gly-CCC-001, and tiRNA-Phe-GAA-003 were upregulated in plasma samples of BC patients, and the levels of circulating tRF-Arg-CCT-017 and tiRNA-Phe-GAA-003 were associated with overall survival and disease-free survival, indicating their potential as biomarkers [98]. By contrast, tRF-Gly-CCC-046, tRF-Tyr-GTA-010 and tRF-Pro-TGG-001 were downregulated in both tissues and sera of early-stage BC patients compared to healthy donors [112]. Six 5’-tRFs were significantly downregulated in plasma of patients with early BC, as well as in cell supernatants, plasma exosomes, and tissues [8]. In another study, tRFs (tRF-1003 and tRF-3001a) showed better diagnostic value than their corresponding miRNAs (miR-4521 and miR-1260a), whose sequences are within the tRFs’ [116]. Koi et al. constructed a diagnostic model using 3 small RNAs: miR-21-5p (3’ addition C), miR-23a-3p and tRF-Lys (TTT), which could achieve high diagnostic accuracy (AUC = 0.92) for BC detection and discriminate BCs as early as stage 0 from controls [109]. In BC patients with T cell exhaustion, low expression of ts-34 or high expression of ts-49 was associated with improved survival, indicating their potential as therapeutic targets to improve patient survival or outcomes of immunotherapies [106].

Triple-negative breast cancer (TNBC) is a refractory subtype of BC with aggressive pathology, chemoresistance and poor overall survival. A big data-driven study uncovered the race disparities of the expression profiles of miRNA isoforms and tRFs between Caucasian and African-American patients with TNBC [97]. Intriguingly, a combination of high levels of tRFs and known miR signatures of BC tumors can distinguish BC-derived extracellular vesicles (EVs) in circulation from other source-derived EVs [178]. Feng et al. analyzed the expression profiles of tRFs in cancer stem cells (CSCs) isolated from TNBC and non-TNBC cell lines and found that tDR-000620 expression level was consistently lower in TNBC CSCs and serum samples, which can independently predict recurrence for TNBC [113]. Besides, two hypoxia-induced tRFs tDR-0009 and tDR-7336 were found mainly involved in maintenance of stem cell population and cellular response to IL-6, thereby facilitating the doxorubicin resistance in TNBC [179]. A recent work found that tRFLys-CTT-010 could interact with glucose-6-phosphatase catalytic subunit (G6PC) to regulate cellular lactate production and glycogen consumption, resulting in enhanced cell proliferation of TNBC [180]. This functional axis may provide novel therapeutic targets for TNBC treatment. For the diagnosis of non-triple negative breast cancer, circulating tDR-7816 expression was reported as a potential biomarker even in early stage [114]. In addition, Honda and colleagues identified the abundant accumulation of SHOT-RNAs only in tissues from estrogen receptor (ER)-positive breast cancer patients, but not in those from triple-negative patients or in normal breast tissues [33]. The generation of SHOT-RNAs were regulated by sex hormone and their receptors through activating ANG cleavage of aminoacylated mature tRNAs, and the resultant accumulation of SHOT-RNAs contributed to BC cell proliferation, thus promoting BC tumorigenesis and tumor growth [33].

Emerging evidence show that tRFs are functional in BC tumorigenesis and progression through versatile mechanisms. Farina et al. identified ts-112 as an oncogenic tRF which was selectively repressed by tumor suppressor RUNX1 to prevent overactive proliferation in breast epithelial cells [181]. Mo and coworkers identified 5’-tiRNA^Val^ and tRF-17-79MP9PP as tumor-suppressors for BC through inhibition of FZD3/Wnt/β-Catenin signaling pathway and THBS1/TGF-β1/Smad3 axis, respectively [110, 111]. Recently, tRF-19-W4PU732S was found overexpressed in BC and promoting BC cell EMT and CSC phenotypes by targeting and inhibiting ribosomal protein-L27A (RPL27A) [104]. The regulatory roles of above-mentioned three tRFs relies on direct sequence complementarity with the 3’UTRs of mRNAs of target proteins [104, 110, 111]. Besides, BC-associated tRFs can also exert various functions by direct interacting with proteins. As mentioned above, in breast cancer Goodarzi et al*.* identified a novel class of i-tRFs sharing common motif that matches the YBX1 recognition sequence [42]. Loss-of-function and gain-of-function studies revealed that these i-tRFs suppressed BC cell growth under serum-starvation, invasion, and metastasis, whereas highly metastatic cells blunted the induction of these i-tRFs to evade this tumor-suppressive pathway [42]. Another tRF named tRF3E derived from the same mature tRNA with i-tRF tRF^Glu^ identified by Goodarzi et al. [42] could interact with nucleolin protein and repressed the translation of p53 mRNA, displaying tumor-suppressor functions [115]. A most recent work also unraveled the direct interaction of nucleolin with another pro-metastatic 5’-tRF^Cys^ through different mechanisms. 5’-tRF^Cys^ could drive the oligomerization of nucleolin with specific metastasis-promoting metabolic transcripts (Mthfd1l and Pafah1b1) to form a ribonucleoprotein complex, thereby protecting these transcripts from exonucleolytic degradation and promoting BC metastasis [105].

### tRFs in GC

GC is one of the most common cancers ranking as the fourth leading cause of cancer death worldwide [182]. To date, increasing numbers of deregulated tRFs involved in GC have been unveiled. Gu et al*.* found that a 5’-tRF, hsa_tsr016141, was significantly overexpressed in GC tissues and serum, with close relation to lymph node metastasis and tumor grade [120]. Besides, the upregulation of tRF-31-U5YKFN8DYDZDD [122] and tRF-23-Q99P9P9NDD [121] were also observed in GC and both significantly associated with lymph node metastasis, TNM stage and vascular invasion. By contrast, tiRNA-5034-GluTTC-2 was downregulated in both GC tumor and plasma, and closely related to tumor size [128]. Other GC-downregulated tRFs (tRF-33-P4R8YP9LON4VDP and tRF-19-3L7L73JD) exhibited significant inhibitory effect on GC cell proliferation and migration while inducing apoptosis and cell cycle arrest [101, 129]. So far, some important clues have been provided by researchers for understanding the molecular mechanisms of GC-related tRFs. Dong et al. showed that a GC-associated tRF tRF-24-V29K9UV3IU impeded GC progression through regulating the Wnt signaling pathway [125], while Zhu and coworkers indicated the inhibition of tRF-5026a on GC progression was associated with the PTEN/PI3K/AKT signaling pathway [99]. The MAPK signaling pathway also participates in the regulatory network orchestrated by GC-related tRFs. For instance, tRF-Val-CAC-016 and tRF-Glu-TTC-027 directly bind to the mRNA 3’UTR of Calcium Voltage-Gated Channel Subunit Alpha1 D (CACNA1d) and transforming growth factor beta 2 (TGFB2) respectively to regulate MAPK signaling pathway and GC progression [123, 124]. In another case, a 3’-tRF tRF-3019a bound with mRNA 3’UTR of tumor suppressor gene F-box protein 47 (FBXO47), resulting in enhanced GC cell proliferation, migration and invasion [118]. Intriguingly, the posttranscriptional gene silencing mediated by some GC-related tRFs is reported to be AGO2-dependant. The tRF-3017A and tRF-24-V29K9UV3IU can function as miRNA-like fragments and bind to AGO2 protein, followed by binding with the 3’UTR of NELL2 and GPR78 mRNAs [117, 126].

In addition to RNAi abilities, GC-related tRF can also interact with RBPs. Recently, Cui and colleagues identified a 3’-tRF named as tRF-Val as a potential oncogene in GC and confirmed the interaction of tRF-Val with the chaperone molecule EEE1A1 [119]. They further unraveled that tRF-Val mediated the transport of EEF1A1 to the nucleus, thus improving the interaction of EEF1A1 and MDM2-p53, which resulted in p53 ubiquitination and blockade of p53 signaling pathways [119]. However, the underlying mechanism of tRF affecting GC progression remains largely unknown and needs further investigation.

### tRFs in CRC

tRF-1001, a tRF-1 type tRF, was identified as a functional RNA fragment which could affect colon cancer cell proliferation. The knockdown of tRF-1001 significantly impaired the growth of HCT116 cells, with specific cell cycle arrest in G2 phase [6]. This inhibitory effect of tRF-1001 depletion was not attributed to a decrease in the corresponding mature tRNA and could be rescued by the introduction of a synthetic tRF-1001 (Me-tRF-1001), indicating that tRF-1001 was necessary in colon cancer cell proliferation [6]. In a tRF signature study, tRF-1001 (termed ts-36 by the authors) showed a two-fold increase in expression level in colon carcinomas but not adenomas [73]. In the same study, only ts-40 showed simultaneous upregulation in both colon carcinomas and adenomas, suggesting its oncogenic role in colon cancer development [73].

With the rapid development of RNA sequencing, increasing CRC-related tRFs have been discovered [132]. Xiong et al. identified 16 significantly differentially expressed tRFs between colon cancer tissues and peri-tumor tissues, including three tRF subtypes (i-tRF, tRF-1, and 5’-tRF) [183]. Zhu et al. successfully built a diagnostic model based on four differentially expressed tRFs (tRF-22-WB86Q3P92, tRF-22-WE8SPOX52, tRF-22-WE8S68L52 and tRF-18-8R1546D2) and a prognostic model based on six significant tRFs (tRF-18-HSRVK7D2, tRF-33-PSQP4PW3FJI0W, tRF-33-PSQP4PW3FJIKW, tRF-18-H9Q867D2, tRF-32-O7M8LOMLQHWU3, and tRF-16-I3FJQSD) from small RNA-seq data to improve CRC diagnosis and recurrence prediction [133]. A recent study investigated the involvement of tRFs in EMT and found that CRC-upregulated tRF-phe-GAA-031 and tRF-VAL-TCA-002 were significantly correlated with distant metastasis and clinical stage [130]. Moreover, higher expressions of these two tRFs were associated with shorter survival for CRC patients, indicating their potentials as prognostic markers [130]. Recently, Tsiakanikas et al*.* reported that high expression of 5’-tiRNA-Pro^TGG^ was not only associated with poor disease-free survival and overall survival, but also could independently predict CRC recurrence [131]. Wu and coworkers proposed plasma 5’-tRF-GlyGCC as a novel diagnostic marker for CRC since its AUC achieved 0.882 and could be improved to 0.926 with the combination of CEA and CA199 [135]. They further found that the increased expression of 5’-tRF-GlyGCC relies on the upregulation of AlkB homolog 3 (ALKBH3), a tRNA demethylase which can enhance tRNA cleavage [135]. Luan et al. found that Dicer1 upregulated the expression of tRF-20-MEJB5Y13 under hypoxic conditions and promoted hypoxia-induced CRC cell invasion and migration [102]. In another study, ANG was found upregulated in CRC tissues and suggested to promote metastasis in CRC via inducing tiRNA (5’-tiRNA-Val) production [134].

The studies on underlying mechanism for the roles of tRFs in CRC are in progress. The GC-downregulated tRF-20-M0NK5Y93 could inhibit CRC cell migration and invasion by binding to the 3’UTR of Claudin-1 mRNA and regulating EMT [184]. In a similar manner, a 17-bp tRF/miR-1280 suppressed CRC cell growth, CSC-like phenotype and metastasis through a direct interaction with JAG2 3’ UTR and subsequent inhibition of Notch signaling pathways [136]. Intriguingly, a recent study revealed that a specific tRNA half, 5’tiRNA-His-GTG, responded to hypoxia via the HIF1α/ANG axis and promoted CRC progression by regulating LATS2 and blocking hippo pathway [12]. Another recent study showed that mimics of tRFs derived non-pathogenic Escherichia coli strains (NPECSs) possess significant cytotoxicity on CRC cells [185]. Among them, EC83 mimic, a double-strand RNA with a 22nt 5’-tRF derived from tRNA-Leu (CAA) as an antisense chain, had highest cytotoxic effect, which can be enhanced by the 2’-O-methylation of the ribose of guanosine (Gm) and resulting stabilization of its tertiary structure [185]. This work may inspire the utilization of NPECS tRFs as potent therapeutic molecules for CRC.

### tRFs in lung cancer

The tRF signatures in lung cancer was uncovered in 2017 and ts-46 and ts-47 are demonstrated as downregulated tRFs with an inhibiting effect on the colony formation ability of H1299 and A549 lung cancer cells [73]. Gu et al. developed a TRY-RNA signature composed of tRFs, rRNA-derived small RNAs, and YRNA-derived small RNAs from human peripheral blood mononuclear cells, which exhibited diagnostic potential for precise discrimination between healthy control, lung cancer and pulmonary tuberculosis [142]. Nowadays, machine learning has been increasingly applied for combined analysis of multiple molecules to achieve higher prediction accuracy. Wang et al. established a model by support vector machine, combining three hub tRFs (tRF-16-L85J3KE, tRF-21-RK9P4P9L0 and tRF-16-PSQP4PE) to predict lung adenocarcinoma with an AUC 0.99 in plasma and 0.92 in tissues [140]. By contrast, the performance of these single tRF to distinguish lung adenocarcinoma in both plasma and tissue was very poor [140]. Another recent study also demonstrated that machine learning diagnostic models constructed with serum RNAs including tRFs, microRNAs, miscellaneous RNAs, and isomiRs could predict lung cancer up to 10 years prior to diagnosis, with a top AUC up to 0.9 [186].

The association and function of single tRFs in lung cancer is partially unveiled. The expression of serum tRF-31-79MP9P9NH57SD was found elevated in non-small cell lung cancer (NSCLC) in relation to clinical stage and the malignancy of lymph node, and significantly decreased after surgery [141], indicating its potential as diagnostic biomarker for NSCLC. Significantly upregulated serum tRF-Leu-CAG was associated with stage progression for NSCLC, and may be involved in regulating AURKA to promote cell proliferation and cell cycle in NSCLC [138]. tsRNA-5001a was found to promote the proliferation of lung adenocarcinoma cells, and its high expression was associated with higher risk of postoperative recurrence [137]. AS-tDR-007872 was recognized as a diagnostic biomarker and a tumor suppressor for NSCLC with inhibitory effect on tumor cell proliferation, invasion, and migration, which is probably induced by targeting BCL2L11 [143]. Recently, Yang et al*.* found that AS-tDR-007333 was significantly upregulated in NSCLC and associated with poor prognosis [139]. They further uncovered that AS-tDR-007333 promoted NSCLC malignancy by activating MED29 through two mechanisms, either interacting with HSPB1, enhancing H3K4me1 and H3K27ac in MED29 promoter and activating MED29 expression, or stimulating the expression of transcription factor ELK4 to bind to MED29 promoter and increase its transcription [139].

### tRFs in leukemia

While studying the miR-4521/3676 cluster in chronic lymphocytic leukemia (CLL), Pekarsky and colleagues found that miR-3676 and miR-4521 are indeed tRFs and involved in cancer pathogenesis [65]. The two tRFs (renamed as “ts-53” and “ts-101”) were down-regulated in CLL and interacted not only with AGO proteins but also with PiwiL2 protein [65]. Later, the same group described a signature of 17 tRFs differentially expressed in CLL, among which only ts-46 and ts-47 are simultaneously downregulated in lung cancer [73]. The Kontos group worked on the role of tRFs in CLL and identified i-tRFs (i-tRF-GlyGCC, i-tRF-GlyCCC and i-tRF-Phe^GAA^) and a 3’-tRF tRF-Leu^AAG/TAG^ as independent prognostic biomarkers for CLL [144, 145, 147, 148]. As described above, tRF-3019 may prime HTLV-1 reverse transcription, thus could act as a novel target to control HTLV-1 infection and HTLV-induced adult T-cell leukemia/lymphoma [92]. Of note, tRFs are also involved in acute myeloid leukemia (AML). The expression of the tDR-Asp family in myelodysplastic syndromes (MDS) patients who later progressed to AML was significantly lower than that in MDS patients who never progressed to AML, which may help predict MDS progression to AML [146].

### tRFs in pancreatic cancer

As reported by the latest WHO cancer statistics, pancreatic cancer accounts for almost as many deaths (466,000) as cases (496,000) owing to its poor survival outcomes [182]. Several studies have indicated that tRF could be applied as potential biomarkers and therapeutic targets for pancreatic cancer. Jin et al. performed high-throughput sequencing in tissues and validated four tRFs including AS-tDR-000064, AS-tDR-000069, AS-tDR-000102, and AS-tDR-001391 as deregulated fragments in pancreatic cancers [149]. Subsequently, a serum two-tRF signature involving tRF-Pro-AGG-004 and tRF-Leu-CAG-002 was indicated as a novel promising biomarker for early pancreatic cancer diagnosis and post-operative survival prediction [151]. In addition, downregulation of tRF-Pro-CGG was observed in pancreatic ductal adenocarcinoma (PDAC) and was associated with short overall survival [153], whereas serum tsRNA-ValTAC-41 was found upregulated in PDAC and its high level predicted poor prognosis [152].

The investigation of underlying mechanisms of pancreatic cancer-associated tRFs is still at its early stage. Sui et al. explored the role of tRF-Leu-AAG in pancreatic cancer cells and found tRF-Leu-AAG promoted cell proliferation, migration, and invasion through directly binding to UPF1 mRNA [150]. In another study, tRF-21-VBY9PYKHD (tRF-21) was identified as a tumor suppressor in PDAC progression, whose generation was inhibited by leukemia inhibitory factor and IL-6 through splicing factor SRSF5 [154]. Further investigations suggested that tRF-21 reduction promoted AKT1/2-mediated heterogeneous nuclear ribonucleoprotein L (hnRNP L) phosphorylation and hnRNP L-DDX17 complex formation, which preferentially spliced Caspase 9 and mH2A1 pre-mRNAs to form Caspase 9b and mH2A1.2, resulting in promoted PDAC cell malignant phenotypes [154].

### tRFs in liver cancer

In addition to the LeuCAG3’tsRNA described before [82], several other tRFs have also been reported in HCC. Zhan and colleagues identified serum mitochondrial tRF-Gln-TTG-006 as a potential biomarker for HCC diagnosis [187]. Recently, a novel five-tRF-based diagnostic model and a seven-tRF-based prognostic model were developed for cancer diagnosis and the overall survival prediction of liver cancer patients [188]. Besides, plasma exosomal tRFs (tRNA-ValTAC-3, tRNAGlyTCC-5, tRNA-ValAAC-5 and tRNA-GluCTC-5) could serve as novel biomarkers for the diagnosis of liver cancer [156]. In terms of mechanisms, an HCC-upregulated tRF Gly-tRF enhanced liver cancer stem cell-like properties and promoted EMT by targeting NDFIP2 and activating the AKT signaling pathway [155]. In addition, Cho et al. identified a distinct set of pre-tRNA 3’ trailer-derived tRFs by analyzing the profiles of small RNAs in Huh7 cells and human liver biopsies, which could sequester nuclear-cytoplasmic shuttling protein La/SSB in the cytoplasm by direct binding with their 3’U-tails, thus negatively regulating La/SSB-dependent viral gene expression [82].

### tRFs in ovarian cancer

Balatti et al. identified that ts-29 was overexpressed more than two folds in cancer samples while ts-3 was upregulated in both ovarian cancer and CLL [73]. The investigation of public RNA-sequencing data from ovarian cancer patients and non-cancer controls revealed that circulating tRFs cover a high proportion (ranging from 2.5–29.4%) of total small RNAs and are not random degradation products in serum [157]. Four differentially expressed tRFs in serum with greatest significance were named as ts1-ts4 (all derived from Gly-tRNA) and further investigated [157]. Among them, ts-3 was optimal which was able to diagnose ovarian cancer from healthy controls with best accuracy (AUC = 0.948), but could not distinguish malignancy from benign [157].

High-grade serous ovarian cancer (HGSOC) accounts for 75% of ovarian cancer cases with highest mortality. The differential expression profile of tRFs was characterized in HGSOC and the tRFs were shown to be involved in multiple pathways such as the AMPK pathway, the glucagon signaling pathway and the insulin signaling pathway [189]. tRF-03357 showed significantly increased expression in HGSOC serum and could promoted SK-OV-3 cell proliferation, migration and invasion partially by downregulating the transcription factor HMBOX1 [159].

tRFs are also associated with the prognosis of ovarian cancer. The elevated expression of i-tRFs derived from tRNAGly^GCC^ in ovarian cancer was correlated with advanced tumor stage and poor overall survival as well as early progression following debulking surgery and platinum-based chemotherapy, which could help with personalized prognosis prediction and clinical decisions [158]. Another 5’-tRF derived from tRNA-Glu-CTC, tRF5-Glu, was identified in ovarian cancer cell [190]. tRF5-Glu could directly bind to the 3’UTR of the Breast Cancer Anti-Estrogen Resistance 3 (BCAR3) mRNA thereby downregulating its expression, resulting in suppressed cancer cell proliferation [190]. Intriguingly, a recent research reported that a tRF mimic (antisense derived from the 5’end of tRNA^His(GUG)^ of Chinese yew) could exhibit comparable anti-cancer activity with taxol on A2780 ovarian cancer cells [191]. Dual-luciferase reporter assay and AGO-RIP assay revealed that the plant-derived tRF-T11 directly targeted the 3’UTR of oncogene TRPA1 mRNA and interacted with AGO2 to suppress ovarian cancer cell growth [191], which sheds light on the development of nature source tRFs as therapeutic molecules.

### tRFs in prostate cancer

In 2009, Lee et al. identified tRF-1001, a tRF-1 derived from the 3’ end of a Ser-TGA tRNA precursor, in ultra-high-throughput sequencing of RNA library constructed from prostate cancer cell line [6]. tRF-1001 was found highly expressed in a wide range of cancer cell lines and correlated with cell proliferation [6]. The generation of tRF-1001 relied on a prostate cancer susceptibility gene, tRNA 3’-endonuclease ELAC2, indicating its specific biological role in prostate cancer [6]. SHOT-RNAs are abundantly expressed in androgen receptor (AR)-positive prostate cancer cell lines LNCaP-FGC cells, but not AR^−^ DU145 and PC-3 cells [33]. Magee and coworkers analyzed the Prostate Cancer datasets of TCGA to obtain profiles of miRNA isoforms and tRFs in prostate cancer and the latter was found to be correlated with different races [96]. tRFs are also involved in chemotherapy resistance. Yang et al. identified that tRF-315 derived from tRNA^Lys^ may protect prostate cancer cells from mitochondrion-dependent apoptosis induced by cisplatin through targeting genes including GADD45A, suggesting that tRF-315 may serve as a therapeutic target or predictive indicator for prostate cancer treatment [192].

### tRFs in bladder cancer

The expression profiles of tRFs were characterized with subsequent bioinformatics analysis in muscle-invasive bladder cancer (MIBC) in a Chinese population [160]. 91 significantly expressed tRFs were identified, among which tiRNA-1:33-Gly-GCC-1, tRF-1:32-Gly-GCC-1, and tRF- + 1:T20-Ser-TGA-1 were validated and likely to participate in the pathophysiological process of MIBC [160]. A 5’-tRF, 5’-tRF-LysCTT, was found significantly deregulated in bladder cancer by in silico analysis of the TCGA-BLCA project [161]. Increased 5’-tRF-LysCTT was associated with aggressive tumor phenotype, early progression of non-muscle-invasive bladder cancer (NMIBC) and poor survival of MIBC [161]. Moreover, the integration of 5’-tRF-LysCTT with the clinically available markers acquired superior risk-stratification specificity and improved prediction of disease progression for bladder cancer, compared with the use of the clinical markers alone [161]. Interestingly, the modification of tRFs could regulate RNA biology in bladder cancer. The N1-methyladenosine (m1A) modification was found TRMT6/61A-dependent and deregulating the targetome of tRFs in urothelial carcinoma of the bladder, resulting in unfolded protein response via 3’-tRF targets [193], which verified the important role of base modification of tRFs in regulating gene silencing.

### tRFs in renal cancer

The RNA sequencing of clear cell renal cell carcinoma (ccRCC) and normal renal tissues showed tRNA reads mapped in the 30–36 nt fraction instead of 73–95 nt, indicating cleavage of tRNAs [162]. Among them, the downregulation of 5’tRNAValAAC was further validated, with an inverse correlation with ccRCC staging and grading [162]. Besides, 5’-halves including 5’-tRNA-Arg-CCT half, 5’-tRNA-Glu-CTC half, 5’-tRNA-Leu-CAG half and 5’-tRNA-Lys-TTT half were also downregulated in the serum and tissue of ccRCC patients [163]. Their lower expression levels were associated with adverse clinicopathological parameters, suggesting the potential of 5’-halves as novel ccRCC biomarkers [163]. Recently, Kazimierczyk and coworkers characterized the expressed tRFs during human renal cell development and found 5’-tRF could replace miR-458 during tumor suppression and regulate MEIS2, FMN1 and CTDSPL2 proteins, resulting in impaired renal function [194]. In silico analysis showed that 5’-tRF could also regulate the activity of renal tumor-associated proteins such as NFIC, GNAO1 and HIPK2 [194].

### tRFs in cervical cancer

In endocervical adenocarcinoma, several hub miRNA/tRFs closely associated with significant clinical phenotypes such as Silva pattern A, gastric subtype and substantial LVSI exhibiting concordant biological function were identified through the weighted gene co-expression network analysis, providing potential biomarkers for personalized medicine of cervical cancer [167]. Shi and colleagues developed panoramic RNA display by overcoming RNA modification aborted sequencing (PANDORA-seq) with a combined enzymatic treatment to remove key RNA modifications that block adapter ligation and reverse transcription, and identified specific expression profiles of abundant tRFs in different tissues and HeLa cells [195]. Besides, a 35 long residue tRF, tRNA^Pro^ 5’ half, was found to be apparent in HeLa cells and could bind to the ribosome, leading to ribosome stalling and the formation of peptidyl-tRNA [34]. In addition, tRF(Gln) was found upregulated in HeLa cells and repressed protein translation without the need for complementary target sites in the mRNA [86]. A 3’-tRF tRNAlys3 was found to associate with AGO2 and target HIV primer binding site in HeLa cells [91]. In another study, two 5’-tRFs, 5’tDR-GlyGCC and 5’tDR-GlnCTG, were revealed to enhance tumor progression of cervical cancer by promoting ribosome assembly and preventing cell apoptosis [52].

### tRFs in other cancers

In addition, tRFs have also been reported in other cancers including esophageal squamous cell carcinoma (ESCC), multiple myeloma (MM), cervical cancer, papillary thyroid cancer (PTC), lymphoma, pharyngeal cancer, laryngeal cancer, head and neck squamous cell carcinoma (HNSCC), and uveal melanoma (UVM), glioma and cholangiocarcinoma. Recently, a novel biomarker signature of saliva-derived exosomal ncRNAs (tRNA-GlyGCC-5 and sRESE) was constructed, which could not only distinguish ESCC patients from healthy controls with 90.5% sensitivity and 94.2% specificity, but also serve as a pre-operative biomarker to identify patients who might benefit from adjuvant therapy [164]. The deregulated tRFs could be potential biomarkers for diagnosing MM from healthy donors [165], distinguishing relapsed/refractory MM from newly diagnosed ones [103], and predicting overall survival of MM patients [166]. The expression profile of tRFs in PTC were characterized and tRF-39-0VL8K87SIRMM12E2, which showed the maximum expression difference between PTC cells and normal cells, was found mostly enriched in the “metabolic pathways” [168]. In PTC, a tRNA half tiRNA-Gly could bind to the U2AF homology motif (UHM) of RBM17 protein and induce the translocation and upregulation of RBM17 by inhibiting its degradation in a ubiquitin/proteasome-dependent way [169]. Furthermore, tiRNA-Gly could induce RBM17-dependent alternative splicing of MAP4K4 mRNA, which led to the phosphorylation of downstream signaling pathways, thereby exerting its oncogenic role in PTC [169]. A 3’-tRF derived from Gly(GCC) tRNA, CU1276, was downregulated in B-cell lymphoma, with its generation regulated by DICER-1 and the miRNA-like ability to associate with all four AGO proteins [170]. Moreover, CU1276 could suppress the proliferation of lymphoma cells and enhance DNA damage response by repressing endogenous RPA1 [170]. The expression profile of tRFs in primary nasopharyngeal carcinoma was identified and two upregulated tRFs (tRF-1:28-Val-CAC-2 and tRF-1:24-Ser-CGA-1-M3) and one downregulated tRF (tRF-55:76-Arg-ACG-1-M2) were validated [171]. Another tRF, tRF-1:30-Lys-CTT-1-M2 was upregulated in hypopharyngeal cancer and identified as an independent risk factor for lung metastasis [100]. In the HNSCC, a multi-marker signature of 3 circulating sncRNAs including miRNAs, tRFs and YRNA-derived small RNAs was identified with significantly deregulated expression in patients’ sera compared with healthy controls, which could contribute to early diagnosis of HNSCC [196]. Furthermore, a specific 5’ tRNA-Val-CAC-2–1 half (reduced in serum of oral squamous cell carcinoma (OSCC) patients and elevated in the tumor tissue) was found to transcriptionally target specific genes involved in the negative regulation of the G1/S transition of the mitotic cell cycle, suggesting its potential as circulating biomarker and target for anticancer therapies of OSCC [174]. In addition, fifteen tRFs were significantly associated with overall survival of HNSCC patients, among which tRF-20-S998LO9D was validated as the top prognostic biomarker [173]. UVM is the most common intraocular tumor in adults with a 50% metastatic rate. By comprehensive characterization of TCGA datasets involving 80 primary UVM tumor samples, the expression profiles of miRNA isoforms and tRFs was described, with close relation to various molecular phenotypes, metastatic disease, and patient survival of UVM [175]. Recently, a 5’-tRF tRF-20-S998LO9D was retrieved through bioinformatics of MINTbase pan-cancer datasets and verified as upregulated in a variety of cancers, including BC, HNSCC, ccRCC, lung squamous cell carcinoma, pheochromocytoma and paraganglioma, and uterine corpus endometrial carcinoma [197]. The elevated expression of tRF-20-S998LO9D indicated poor prognosis in a variety of cancers and could be a potential pan-cancer biomarker [197].

## Extracellular tRFs for cancer diagnosis and prognosis

**Fig. 3:**
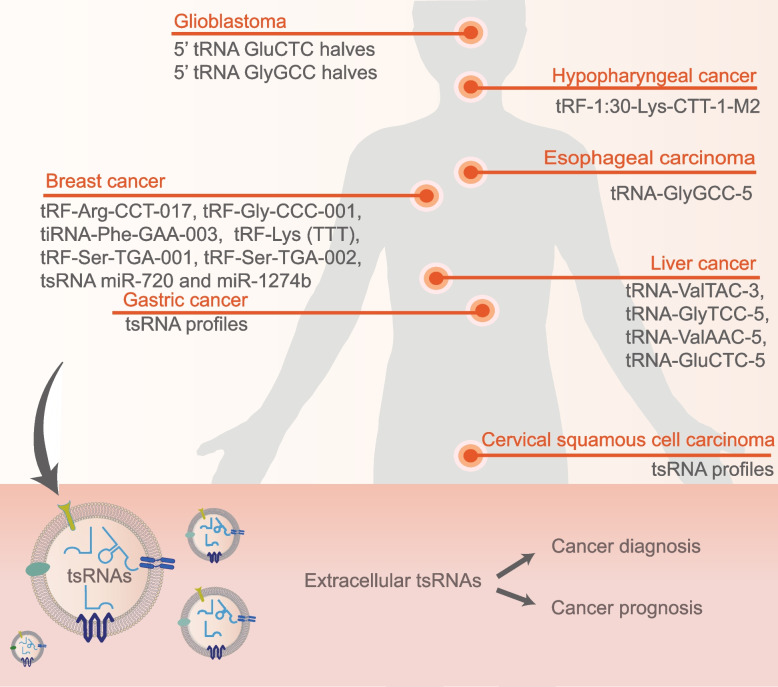
Extracellular tRFs involved in cancers. Certain extracellular tRFs derived from peripheral blood or salivary are aberrantly expressed and associated with cancers including glioblastoma, hypopharyngeal cancer, breast cancer, esophageal carcinoma, liver cancer, gastric cancer, and cervical squamous cell carcinoma. Detection of these extracellular tRFs contributes to early cancer diagnosis and prognosis prediction

Emerging evidence have shown that tRFs encapsulated in EVs may play key roles in cellular process regulation and intercellular communication, providing promising biomarkers for cancer liquid biopsy. The extracellular tRFs involved in cancer are illustrated in Fig. 3.

High-throughput sequencing and verification in a large cohort of 120 patients with BC and 112 healthy controls identified tRF-Arg-CCT-017, tRF-Gly-CCC-001, and tiRNA-Phe-GAA-003 as circulating diagnostic markers of BC [98]. These 3 tRFs showed consistent upregulation in BC plasma and exosomes with obvious correlations observed in tRF-Gly-CCC-001 and tiRNA-Phe-GAA-003, indicating their existence in plasma exosomes [98]. A tRF-Lys (TTT) was found elevated in serum of BC patients, along with miR-21-5p and miR-23a-3p [109]. However, while miR-21-5p and miR-23a-3p showed consistent abundance in serum EVs of BC patients, tRF-Lys (TTT) were not aberrantly included in EVs [109]. Similarly, in another study, only two (tRF-Ser-TGA-001 and tRF-Ser-TGA-002) out of six tRFs were found consistently downregulated in exosomes and plasma samples of patients with early breast cancer compared with healthy controls [8]. One explanation to the inconsistency is the potential existence of other circulating forms of tRFs, which needs further investigation. The “miRNA-like” tRNA fragments miR-720 and miR-1274b were found overexpressed in MCF-7 cells and the expression was greatly amplified in MCF-7 EVs [178]. Moreover, the combined analysis of these tRFs and known miR signatures could distinguish breast tumor-derived EVs from EVs originated from other cell sources [178]. Likewise, Tosar and colleagues sequenced intracellular small RNAs of breast epithelial cell lines (MCF-7 and MCF-10A) and extracellular fractions enriched in microvesicles, exosomes and ribonucleoprotein complexes [198]. It turned out that 5’-tRNA halves of 31–33 nt were significantly enriched in the extracellular space and constituted part of ~ 45 kDa ribonucleoprotein complexes [198].

A deep sequencing of exosomal RNAs revealed that tRFs with 20–40 nt length accounted for less than 10% of all annotated small RNAs derived from human gastric cancer cell lines [199]. Tong and coworkers investigated the profiles of extracellular RNAs derived from cell cultures of HPV-induced HNSCC and cervical squamous cell carcinoma (CSCC) cells [200]. The tRNAs were much more enriched in exosomes and MVs (31% and 34%) than in source cells (3%), with 5’-tRFs and 5’-halves representing 90% of total tRNA reads in extracellular RNAs [200]. The exosomes and MVs shared similar characteristics in tRNA composition with GluCTC being the most overrepresented tRNA (32.6% and 29.9%, respectively), different from source cells in which GlyGCC being most overrepresented [200]. Another compositional characterization of extracellular RNA complexes associated with microvesicles, EVs, and RNPs was performed by Wei et al*.* in human glioma stem cells (GSCs) [201]. Remarkable enrichment of ANG and 5’tRNA halves from specific tRNAs such as GluCTC and GlyGCC were observed in GSC-derived exosomes, suggesting the occurrence of tRNA cleavage in exosomes [201]. Patients with liver cancer exhibited significantly higher tRNA-ValTAC-3, tRNAGlyTCC-5, tRNA-ValAAC-5 and tRNA-GluCTC-5 levels in their plasma exosomes than healthy controls, demonstrating the diagnostic potential of these tRFs for liver cancer [156]. In addition, tRF-1:30-Lys-CTT-1-M2 was identified as overexpressed in plasma EVs of hypopharyngeal cancer patients and further validated in newly diagnosed cancer patients. Furthermore, a significant upregulation of tRF-1:30-Lys-CTT-1-M2 was presented in the lung metastatic hypopharyngeal cancer patients compared with the non- metastatic patients, which was considered as an independent risk factor for the metastasis of hypopharyngeal cancer [100].

In addition to exosomes derived from peripheral blood, salivary exosomes also contain cancer-associated tRFs. A recent study identified differentially expressed exosomal small RNAs by RNA sequencing of salivary exosomes obtained from 3 ESCC patients and 3 healthy controls in a pilot study and further validated in discovery cohort [164]. The developed tRF-based signature (tRNA-GlyGCC-5 and sRESE) could discriminate ESCC patients from the controls with high sensitivity and specificity and predict overall survival [164]. In particular, it could also predict preoperative patients who would benefit from adjuvant therapy [164]. Therefore, salivary exosomal tRFs may represent non-invasive, more convenient, and reliable biomarkers for cancer diagnosis and prognosis. Emerging evidence have shown that tRFs encapsulated in EVs may play key roles in cellular process regulation and intercellular communication, providing promising biomarkers for cancer liquid biopsy.

## Conclusion and perspectives

The development and application of high-throughput transcriptome sequencing has led to the ever-growing discovery of novel tRFs. Further investigations of tRFs for their biological functions and underlying mechanisms are in progress, highlighting the key roles of tRFs in various diseases by diverse activities such as nascent RNA silencing, transcription inhibition, epigenetic gene silencing, and translation regulation. Herein, we reviewed the current status of tRF research in cancer. The tRFs can be divided into tRNA halves and tRFs, including canonical fragments with 5’ or 3’ termini from mature tRNAs, and uncanonical ones such as i-tRFs, tRF-1 s and 5’leader-5’exon tRFs. Meanwhile, various modifications of tRNAs regulate tRF biogenesis, and influence the stability and function of tRFs. The tRFs play critical roles in biological processes including nascent RNA silencing, transcriptional inhibition, post-transcriptional gene silencing, translational regulation, inhibition of apoptosis, regulation of reverse transcription, and regulation of chromatin accessibility. The ubiquitous expression of tRFs has been reported in various cancers, with their function and regulatory mechanism partially unveiled. In addition, we summarized the extracellular tRFs relevant with several cancer types such as breast cancer, liver cancer, glioblastoma, esophageal carcinoma and hypopharyngeal cancer, hoping to provide promising biomarker candidates for cancer liquid biopsy.

However, there are still several limitations in the field of tRF research. First, the nomenclature of tRF is debated, resulting in inconsistency in publications and confusion of readers. Since one tRF can originate from different isodecoders of different tRNAs, the MINTbase database employs the “License Plates” nomenclature for naming tRFs (e.g., tRF-24-7SIRMM12E2), which is totally based on nucleotide sequence to eliminate ambiguities for the same exact molecule [95]. Some sequencing results use tDRs to address tRFs (e.g., AS-tDR-000064), while others follow other nomenclatures (e.g., tRF-Val-CAC-016) [123, 149]. Moreover, some tRFs are named by the order of discovery or the preference of authors (e.g., tRF-1001 is called ts-36 in another paper [6]), making searching through and comparison of different publications more difficult [6, 73]. Therefore, a clear and standardized nomenclature of tRF is urgently needed. Secondly, the knowledge we’ve gained of tRFs is far from satisfaction. Although the dysregulation of some tRFs has been revealed in cancer, a vast number of tRFs are not identified yet, with their biological functions and underlying mechanisms remaining enigmatic. Further comprehensive investigations are urgently required. Thirdly, there is a long way to go to utilize tRF in clinic as biomarkers for cancer. Although some researches emphasized the potential of tRF for cancer diagnosis, few of them could reach excellent specificity and sensitivity without combined analysis of conventional biomarkers. More importantly, the detection of tRFs mainly relies on high-throughput sequencing and stem loop RT-PCR, which are too costly or complicated for large scale detection. More attempts should be made for developing appropriate tRF testing methods. Fourthly, the strategy of current studies using synthesized tRFs to investigate their biological activities may be defective. Due to the incomplete knowledge of tRFs, the synthesized tRFs generally lack modification to exactly mimic endogenous tRFs. Without certain modifications, the synthesized tRFs may exert different spatial structures and resulting in totally varied activities. In fact, the pretreatment of tRF sequencing removing modifications for further detection might have altered the original structure of some tRFs and even led to RNA degradation, making the sequencing results unfaithful. Thus, more efforts should be made to either obtain adequate highly-purified endogenous tRFs, or figure out their exact modifications and spatial structures for precise mimicry.

In conclusion, increasing tRFs with various biological functions are being identified. Current results from preliminary studies reveal the significant association of dysregulation of tRFs with cancer progression, yet with their regulatory networks largely unraveled. Future in-depth investigations are urgently needed for better understanding of tRFs in cancer progression and providing potent therapeutic molecules or qualified biomarkers for cancer diagnosis and prognosis.

## Abbreviations

aa-tRNA: Aminoacyl-tRNA
ALKBH3: AlkB homolog 3
AML: Acute myeloid leukemia
ANG: Angiogenin
AR: Androgen receptor
BC: Breast cancer
BCAR3: Breast cancer anti-estrogen resistance 3
BCDIN3D: Bicoid interacting 3 domain containing RNA methyltransferase
CACNA1d: Calcium voltage-gated channel subunit alpha1 D
ccRCC: Clear cell renal cell carcinoma
CLL: Chronic lymphocytic leukemia
CRC: Colorectal cancer
CSCC: Cervical squamous cell carcinoma
CSC: Cancer stem cell
Cyt c: Cytochrome c
DNMT2: DNA methyltransferase 2
ELAC2: ElaC ribonuclease Z 2
ER: Estrogen receptor
ERV: Endogenous retrovirus
ESCC: Esophageal squamous cell cancer
EV: Extracellular vesicle
FBXO47: F-box protein 47
G4: G-quadruplex
G6PC: Glucose-6-phosphatase catalytic subunit
GC: Gastric cancer
GSC: Glioma stem cell HCC hepatocellular carcinoma
hESC: Human embryonic stem cell
HGSOC: High-grade serous ovarian cancer
hnRNP L: Heterogeneous nuclear ribonucleoprotein L
HNSCC: Head and neck squamous cell carcinoma
HP1β: Heterochromatin protein 1β
HTLV-1: Human T cell leukemia virus type 1
MDS: Myelodysplastic syndrome
MEF: Mouse embryonic fibroblast
MIBC: Muscle-invasive bladder cancer
MM: Multiple myeloma
MSC: Multisynthetase complex
mTOG: Mini TOG
ncRNA: Non-coding RNA
NMIBC: Non-muscle-invasive bladder cancer
NPECS: Non-pathogenic Escherichia coli strain
NSCLC: Non-small cell lung cancer
NSUN2: NOP2/Sun RNA methyltransferase 2
OSCC: Oral squamous cell carcinoma
PABPC1: Polyadenylate-binding protein 1
PANDORA-seq: Panoramic RNA display by overcoming RNA modification aborted sequencing
PAR-CLIP: Photoactivatable-ribonucleoside-enhanced crosslinking and immunoprecipitation
PBS: Primer binding site
PDAC: Pancreatic ductal adenocarcinoma
piRNA: PIWI-interacting RNA
PNK: Polynucleotide kinase
pre-tRNA: Precursor tRNA
PTC: Papillary thyroid cancer
PUS: Pseudouridine synthase
PUS7: Pseudouridine synthetase
Q: Queuosine
RBP: RNA-binding protein
RISC: RNA-induced silencing complex
RPL27A: Ribosomal protein-L27A
SG: Stress granule
SHOT-RNA: Sex hormone-dependent tRNA-derived RNA
td-piR (Glu): tRNA-Glu-derived piRNA
TE: Transposable element
TGFB2: Transforming growth factor beta 2
tiRNA: tRNA-derived stress-inducible RNA
TNBC: Triple-negative breast cancer
TOG: Terminal oligoguanine
tRF: tRNA-derived fragment
TRMT2A: tRNA methyltransferase 2 homolog A
tRNA: Transfer RNA
UHM: U2AF homology motif
UVM: Uveal melanoma
YBX1 or YB-1: Y box binding protein 1
Ψ: Pseudouridylation

## References

[1] Kirchner S, Ignatova Z (2015). Emerging roles of tRNA in adaptive translation, signalling dynamics and disease. Nat Rev Genet.

[2] Goodarzi H, Nguyen HCB, Zhang S, Dill BD, Molina H, Tavazoie SF (2016). Modulated Expression of Specific tRNAs Drives Gene Expression and Cancer Progression. Cell.

[3] Ishimura R, Nagy G, Dotu I, Zhou H, Yang XL, Schimmel P (2014). RNA function. Ribosome stalling induced by mutation of a CNS-specific tRNA causes neurodegeneration. Science..

[4] Liu F, Clark W, Luo G, Wang X, Fu Y, Wei J (2016). ALKBH1-Mediated tRNA Demethylation Regulates Translation. Cell.

[5] Chionh YH, McBee M, Babu IR, Hia F, Lin W, Zhao W (2016). tRNA-mediated codon-biased translation in mycobacterial hypoxic persistence. Nat Commun.

[6] Lee YS, Shibata Y, Malhotra A, Dutta A (2009). A novel class of small RNAs: tRNA-derived RNA fragments (tRFs). Genes Dev.

[7] Godoy PM, Bhakta NR, Barczak AJ, Cakmak H, Fisher S, MacKenzie TC (2018). Large Differences in Small RNA Composition Between Human Biofluids. Genes (Basel).

[8] Wang J, Ma G, Li M, Han X, Xu J, Liang M (2020). Plasma tRNA Fragments Derived from 5' Ends as Novel Diagnostic Biomarkers for Early-Stage Breast Cancer. Mol Ther Nucleic Acids.

[9] Di Fazio A, Schlackow M, Pong SK, Alagia A, Gullerova M (2022). Dicer dependent tRNA derived small RNAs promote nascent RNA silencing. Nucleic Acids Res.

[10] Zhang Y, Ren L, Sun X, Zhang Z, Liu J, Xin Y (2021). Angiogenin mediates paternal inflammation-induced metabolic disorders in offspring through sperm tsRNAs. Nat Commun.

[11] Fagan SG, Helm M, Prehn JHM (2021). tRNA-derived fragments: A new class of non-coding RNA with key roles in nervous system function and dysfunction. Prog Neurobiol.

[12] Tao EW, Wang HL, Cheng WY, Liu QQ, Chen YX, Gao QY (2021). A specific tRNA half, 5'tiRNA-His-GTG, responds to hypoxia via the HIF1α/ANG axis and promotes colorectal cancer progression by regulating LATS2. J Exp Clin Cancer Res.

[13] Hoagland MB, Stephenson ML, Scott JF, Hecht LI, Zamecnik PC (1958). A soluble ribonucleic acid intermediate in protein synthesis. J Biol Chem.

[14] Phan HD, Lai LB, Zahurancik WJ, Gopalan V (2021). The many faces of RNA-based RNase P, an RNA-world relic. Trends Biochem Sci.

[15] Phizicky EM, Hopper AK (2010). tRNA biology charges to the front. Genes Dev.

[16] Abelson J, Trotta CR, Li H (1998). tRNA splicing. J Biol Chem.

[17] Sprinzl M, Cramer F (1979). The -C-C-A end of tRNA and its role in protein biosynthesis. Prog Nucleic Acid Res Mol Biol.

[18] Deutscher MP (1972). Reactions at the 3' terminus of transfer ribonucleic acid. 3. Catalytic properties of two purified rabbit liver transfer ribonucleic acid nucleotidyl transferases. J Biol Chem..

[19] Holley RW, Apgar J, Everett GA, Madison JT, Marquisee M, Merrill SH (1965). STRUCTURE OF A RIBONUCLEIC ACID. Science.

[20] Schimmel P, Ribas de Pouplana L (1995). Transfer RNA: from minihelix to genetic code. Cell..

[21] Pliatsika V, Loher P, Telonis AG, Rigoutsos I (2016). MINTbase: a framework for the interactive exploration of mitochondrial and nuclear tRNA fragments. Bioinformatics.

[22] Anderson P, Ivanov P (2014). tRNA fragments in human health and disease. FEBS Lett.

[23] Fu H, Feng J, Liu Q, Sun F, Tie Y, Zhu J (2009). Stress induces tRNA cleavage by angiogenin in mammalian cells. FEBS Lett.

[24] Blanco S, Dietmann S, Flores JV, Hussain S, Kutter C, Humphreys P (2014). Aberrant methylation of tRNAs links cellular stress to neuro-developmental disorders. Embo j.

[25] Kfoury YS, Ji F, Mazzola M, Sykes DB, Scherer AK, Anselmo A (2021). tiRNA signaling via stress-regulated vesicle transfer in the hematopoietic niche. Cell Stem Cell.

[26] Elkordy A, Mishima E, Niizuma K, Akiyama Y, Fujimura M, Tominaga T (2018). Stress-induced tRNA cleavage and tiRNA generation in rat neuronal PC12 cells. J Neurochem.

[27] Taft RJ, Simons C, Nahkuri S, Oey H, Korbie DJ, Mercer TR (2010). Nuclear-localized tiny RNAs are associated with transcription initiation and splice sites in metazoans. Nat Struct Mol Biol.

[28] Thompson DM, Parker R (2009). The RNase Rny1p cleaves tRNAs and promotes cell death during oxidative stress in Saccharomyces cerevisiae. J Cell Biol.

[29] Akat KM, Lee YA, Hurley A, Morozov P, Max KE, Brown M (2019). Detection of circulating extracellular mRNAs by modified small-RNA-sequencing analysis. JCI Insight.

[30] Su Z, Kuscu C, Malik A, Shibata E, Dutta A (2019). Angiogenin generates specific stress-induced tRNA halves and is not involved in tRF-3-mediated gene silencing. J Biol Chem.

[31] Elbarbary RA, Takaku H, Uchiumi N, Tamiya H, Abe M, Takahashi M (2009). Modulation of gene expression by human cytosolic tRNase Z(L) through 5'-half-tRNA. PLoS ONE.

[32] Wang Q, Lee I, Ren J, Ajay SS, Lee YS, Bao X (2013). Identification and functional characterization of tRNA-derived RNA fragments (tRFs) in respiratory syncytial virus infection. Mol Ther.

[33] Honda S, Loher P, Shigematsu M, Palazzo JP, Suzuki R, Imoto I (2015). Sex hormone-dependent tRNA halves enhance cell proliferation in breast and prostate cancers. Proc Natl Acad Sci U S A.

[34] Gonskikh Y, Gerstl M, Kos M, Borth N, Schosserer M, Grillari J (2020). Modulation of mammalian translation by a ribosome-associated tRNA half. RNA Biol.

[35] Kumar P, Anaya J, Mudunuri SB, Dutta A (2014). Meta-analysis of tRNA derived RNA fragments reveals that they are evolutionarily conserved and associate with AGO proteins to recognize specific RNA targets. BMC Biol.

[36] Cole C, Sobala A, Lu C, Thatcher SR, Bowman A, Brown JW (2009). Filtering of deep sequencing data reveals the existence of abundant Dicer-dependent small RNAs derived from tRNAs. RNA.

[37] Babiarz JE, Ruby JG, Wang Y, Bartel DP, Blelloch R (2008). Mouse ES cells express endogenous shRNAs, siRNAs, and other Microprocessor-independent, Dicer-dependent small RNAs. Genes Dev.

[38] Li Z, Ender C, Meister G, Moore PS, Chang Y, John B (2012). Extensive terminal and asymmetric processing of small RNAs from rRNAs, snoRNAs, snRNAs, and tRNAs. Nucleic Acids Res.

[39] Kumar P, Mudunuri SB, Anaya J, Dutta A (2015). tRFdb: a database for transfer RNA fragments. Nucleic Acids Res..

[40] Telonis AG, Loher P, Honda S, Jing Y, Palazzo J, Kirino Y (2015). Dissecting tRNA-derived fragment complexities using personaized transcriptomes reveals novel fragment classes and unexpected dependencies. Oncotarget.

[41] Olvedy M, Scaravilli M, Hoogstrate Y, Visakorpi T, Jenster G, Martens-Uzunova ES (2016). A comprehensive repertoire of tRNA-derived fragments in prostate cancer. Oncotarget.

[42] Goodarzi H, Liu X, Nguyen HC, Zhang S, Fish L, Tavazoie SF (2015). Endogenous tRNA-Derived Fragments Suppress Breast Cancer Progression via YBX1 Displacement. Cell.

[43] Telonis AG, Loher P, Magee R, Pliatsika V, Londin E, Kirino Y (2019). tRNA Fragments Show Intertwining with mRNAs of Specific Repeat Content and Have Links to Disparities. Can Res.

[44] Haussecker D, Huang Y, Lau A, Parameswaran P, Fire AZ, Kay MA (2010). Human tRNA-derived small RNAs in the global regulation of RNA silencing. RNA.

[45] Kim HK, Yeom JH, Kay MA (2020). Transfer RNA-Derived Small RNAs: Another Layer of Gene Regulation and Novel Targets for Disease Therapeutics. Mol Ther.

[46] Sobala A, Hutvagner G (2011). Transfer RNA-derived fragments: origins, processing, and functions. WIREs RNA.

[47] Hanada T, Weitzer S, Mair B, Bernreuther C, Wainger BJ, Ichida J (2013). CLP1 links tRNA metabolism to progressive motor-neuron loss. Nature.

[48] Lyko F (2018). The DNA methyltransferase family: a versatile toolkit for epigenetic regulation. Nat Rev Genet.

[49] Zhang Y, Zhang X, Shi J, Tuorto F, Li X, Liu Y (2018). Dnmt2 mediates intergenerational transmission of paternally acquired metabolic disorders through sperm small non-coding RNAs. Nat Cell Biol.

[50] Pereira M, Ribeiro DR, Pinheiro MM, Ferreira M, Kellner S, Soares AR (2021). m(5)U54 tRNA Hypomodification by Lack of TRMT2A Drives the Generation of tRNA-Derived Small RNAs. Int J Mol Sci.

[51] Reinsborough CW, Ipas H, Abell NS, Nottingham RM, Yao J, Devanathan SK (2019). BCDIN3D regulates tRNAHis 3' fragment processing. PLoS Genet.

[52] Chen Z, Qi M, Shen B, Luo G, Wu Y, Li J (2019). Transfer RNA demethylase ALKBH3 promotes cancer progression via induction of tRNA-derived small RNAs. Nucleic Acids Res.

[53] Rashad S, Han X, Sato K, Mishima E, Abe T, Tominaga T (2020). The stress specific impact of ALKBH1 on tRNA cleavage and tiRNA generation. RNA Biol.

[54] Guzzi N, Cieśla M, Ngoc PCT, Lang S, Arora S, Dimitriou M (2018). Pseudouridylation of tRNA-Derived Fragments Steers Translational Control in Stem Cells. Cell.

[55] Fergus C, Barnes D, Alqasem MA, Kelly VP (2015). The queuine micronutrient: charting a course from microbe to man. Nutrients.

[56] Wang X, Matuszek Z, Huang Y, Parisien M, Dai Q, Clark W (2018). Queuosine modification protects cognate tRNAs against ribonuclease cleavage. RNA.

[57] Ehrenhofer-Murray AE (2017). Cross-Talk between Dnmt2-Dependent tRNA Methylation and Queuosine Modification. Biomolecules.

[58] Müller M, Hartmann M, Schuster I, Bender S, Thüring KL, Helm M (2015). Dynamic modulation of Dnmt2-dependent tRNA methylation by the micronutrient queuine. Nucleic Acids Res.

[59] Chan CM, Zhou C, Huang RH (2009). Reconstituting bacterial RNA repair and modification in vitro. Science.

[60] Vitali P, Kiss T (2019). Cooperative 2'-O-methylation of the wobble cytidine of human elongator tRNA(Met)(CAT) by a nucleolar and a Cajal body-specific box C/D RNP. Genes Dev.

[61] Angelova MT, Dimitrova DG, Da Silva B, Marchand V, Jacquier C, Achour C (2020). tRNA 2'-O-methylation by a duo of TRM7/FTSJ1 proteins modulates small RNA silencing in Drosophila. Nucleic Acids Res.

[62] Galvanin A, Vogt L-M, Grober A, Freund I, Ayadi L, Bourguignon-Igel V (2020). Bacterial tRNA 2'-O-methylation is dynamically regulated under stress conditions and modulates innate immune response. Nucleic Acids Res.

[63] Kalantari R, Chiang CM, Corey DR (2016). Regulation of mammalian transcription and splicing by Nuclear RNAi. Nucleic Acids Res.

[64] Gagnon KT, Li L, Chu Y, Janowski BA, Corey DR (2014). RNAi factors are present and active in human cell nuclei. Cell Rep.

[65] Pekarsky Y, Balatti V, Palamarchuk A, Rizzotto L, Veneziano D, Nigita G (2016). Dysregulation of a family of short noncoding RNAs, tsRNAs, in human cancer. Proc Natl Acad Sci U S A.

[66] Genzor P, Cordts SC, Bokil NV, Haase AD (2019). Aberrant expression of select piRNA-pathway genes does not reactivate piRNA silencing in cancer cells. Proc Natl Acad Sci USA.

[67] Zhang X, He X, Liu C, Liu J, Hu Q, Pan T (2016). IL-4 Inhibits the Biogenesis of an Epigenetically Suppressive PIWI-Interacting RNA To Upregulate CD1a Molecules on Monocytes/Dendritic Cells. J Immunol.

[68] Chen Q, Yan M, Cao Z, Li X, Zhang Y, Shi J (2016). Sperm tsRNAs contribute to intergenerational inheritance of an acquired metabolic disorder. Science.

[69] Karaiskos S, Naqvi AS, Swanson KE, Grigoriev A (2015). Age-driven modulation of tRNA-derived fragments in Drosophila and their potential targets. Biol Direct.

[70] Kuscu C, Kumar P, Kiran M, Su Z, Malik A, Dutta A (2018). tRNA fragments (tRFs) guide Ago to regulate gene expression post-transcriptionally in a Dicer-independent manner. RNA.

[71] Martinez G, Choudury SG, Slotkin RK (2017). tRNA-derived small RNAs target transposable element transcripts. Nucleic Acids Res.

[72] Couvillion MT, Bounova G, Purdom E, Speed TP, Collins K (2012). A Tetrahymena Piwi bound to mature tRNA 3' fragments activates the exonuclease Xrn2 for RNA processing in the nucleus. Mol Cell.

[73] Balatti V, Nigita G, Veneziano D, Drusco A, Stein GS, Messier TL (2017). tsRNA signatures in cancer. Proc Natl Acad Sci U S A.

[74] Wang JZ, Zhu H, You P, Liu H, Wang WK, Fan X (2022). Upregulated YB-1 protein promotes glioblastoma growth through a YB-1/CCT4/mLST8/mTOR pathway. J Clin Invest.

[75] Budkina K, El Hage K, Clément M-J, Desforges B, Bouhss A, Joshi V (2021). YB-1 unwinds mRNA secondary structures in vitro and negatively regulates stress granule assembly in HeLa cells. Nucleic Acids Res.

[76] Krishna S, Yim DG, Lakshmanan V, Tirumalai V, Koh JL, Park JE (2019). Dynamic expression of tRNA-derived small RNAs define cellular states. EMBO Rep.

[77] Ivanov P, Emara MM, Villen J, Gygi SP, Anderson P (2011). Angiogenin-induced tRNA fragments inhibit translation initiation. Mol Cell.

[78] Lyons SM, Achorn C, Kedersha NL, Anderson PJ, Ivanov P (2016). YB-1 regulates tiRNA-induced Stress Granule formation but not translational repression. Nucleic Acids Res.

[79] Ivanov P, O'Day E, Emara MM, Wagner G, Lieberman J, Anderson P (2014). G-quadruplex structures contribute to the neuroprotective effects of angiogenin-induced tRNA fragments. Proc Natl Acad Sci U S A.

[80] Lyons SM, Gudanis D, Coyne SM, Gdaniec Z, Ivanov P (2017). Identification of functional tetramolecular RNA G-quadruplexes derived from transfer RNAs. Nat Commun.

[81] Lyons SM, Kharel P, Akiyama Y, Ojha S, Dave D, Tsvetkov V (2020). eIF4G has intrinsic G-quadruplex binding activity that is required for tiRNA function. Nucleic Acids Res.

[82] Kim HK, Fuchs G, Wang S, Wei W, Zhang Y, Park H (2017). A transfer-RNA-derived small RNA regulates ribosome biogenesis. Nature.

[83] Kim HK, Xu J, Chu K, Park H, Jang H, Li P (2019). A tRNA-Derived Small RNA Regulates Ribosomal Protein S28 Protein Levels after Translation Initiation in Humans and Mice. Cell Rep.

[84] Liu Z, Kim HK, Xu J, Jing Y, Kay MA (2021). The 3'tsRNAs are aminoacylated: Implications for their biogenesis. PLoS Genet.

[85] Keam SP, Sobala A, Ten Have S, Hutvagner G (2017). tRNA-Derived RNA Fragments Associate with Human Multisynthetase Complex (MSC) and Modulate Ribosomal Protein Translation. J Proteome Res.

[86] Sobala A, Hutvagner G (2013). Small RNAs derived from the 5' end of tRNA can inhibit protein translation in human cells. RNA Biol.

[87] Bevilacqua E, Wang X, Majumder M, Gaccioli F, Yuan CL, Wang C (2010). eIF2alpha phosphorylation tips the balance to apoptosis during osmotic stress. J Biol Chem.

[88] Saikia M, Jobava R, Parisien M, Putnam A, Krokowski D, Gao XH (2014). Angiogenin-cleaved tRNA halves interact with cytochrome c, protecting cells from apoptosis during osmotic stress. Mol Cell Biol.

[89] Slotkin RK, Martienssen R (2007). Transposable elements and the epigenetic regulation of the genome. Nat Rev Genet.

[90] Schorn AJ, Gutbrod MJ, LeBlanc C, Martienssen R (2017). LTR-Retrotransposon Control by tRNA-Derived Small RNAs. Cell.

[91] Yeung ML, Bennasser Y, Watashi K, Le SY, Houzet L, Jeang KT (2009). Pyrosequencing of small non-coding RNAs in HIV-1 infected cells: evidence for the processing of a viral-cellular double-stranded RNA hybrid. Nucleic Acids Res.

[92] Ruggero K, Guffanti A, Corradin A, Sharma VK, De Bellis G, Corti G (2014). Small noncoding RNAs in cells transformed by human T-cell leukemia virus type 1: a role for a tRNA fragment as a primer for reverse transcriptase. J Virol.

[93] Sharma U, Conine CC, Shea JM, Boskovic A, Derr AG, Bing XY (2016). Biogenesis and function of tRNA fragments during sperm maturation and fertilization in mammals. Science.

[94] Boskovic A, Bing XY, Kaymak E, Rando OJ (2020). Control of noncoding RNA production and histone levels by a 5' tRNA fragment. Genes Dev.

[95] Pliatsika V, Loher P, Magee R, Telonis AG, Londin E, Shigematsu M (2018). MINTbase v2.0: a comprehensive database for tRNA-derived fragments that includes nuclear and mitochondrial fragments from all The Cancer Genome Atlas projects. Nucleic Acids Res..

[96] Magee RG, Telonis AG, Loher P, Londin E, Rigoutsos I (2018). Profiles of miRNA Isoforms and tRNA Fragments in Prostate Cancer. Sci Rep.

[97] Telonis AG, Rigoutsos I (2018). Race Disparities in the Contribution of miRNA Isoforms and tRNA-Derived Fragments to Triple-Negative Breast Cancer. Cancer Res.

[98] Wang J, Ma G, Ge H, Han X, Mao X, Wang X (2021). Circulating tRNA-derived small RNAs (tsRNAs) signature for the diagnosis and prognosis of breast cancer. NPJ Breast Cancer.

[99] Zhu L, Li Z, Yu X, Ruan Y, Shen Y, Shao Y (2021). The tRNA-derived fragment 5026a inhibits the proliferation of gastric cancer cells by regulating the PTEN/PI3K/AKT signaling pathway. Stem Cell Res Ther.

[100] Xi J, Zeng Z, Li X, Zhang X, Xu J (2021). Expression and Diagnostic Value of tRNA-Derived Fragments Secreted by Extracellular Vesicles in Hypopharyngeal Carcinoma. Onco Targets Ther.

[101] Shen Y, Yu X, Ruan Y, Li Z, Xie Y, Yan Z (2021). Global profile of tRNA-derived small RNAs in gastric cancer patient plasma and identification of tRF-33-P4R8YP9LON4VDP as a new tumor suppressor. Int J Med Sci.

[102] Luan N, Mu Y, Mu J, Chen Y, Ye X, Zhou Q (2021). Dicer1 Promotes Colon Cancer Cell Invasion and Migration Through Modulation of tRF-20-MEJB5Y13 Expression Under Hypoxia. Front Genet.

[103] Xu C, Liang T, Zhang F, Liu J, Fu Y (2021). tRNA-derived fragments as novel potential biomarkers for relapsed/refractory multiple myeloma. BMC Bioinformatics.

[104] Zhang Z, Liu Z, Zhao W, Zhao X, Tao Y (2022). tRF-19-W4PU732S promotes breast cancer cell malignant activity by targeting inhibition of RPL27A (ribosomal protein-L27A). Bioengineered.

[105] Liu X, Mei W, Padmanaban V, Alwaseem H, Molina H, Passarelli MC (2022). A pro-metastatic tRNA fragment drives Nucleolin oligomerization and stabilization of its bound metabolic mRNAs. Mol Cell.

[106] Shan N, Li N, Dai Q, Hou L, Yan X, Amei A (2020). Interplay of tRNA-Derived Fragments and T Cell Activation in Breast Cancer Patient Survival. Cancers (Basel)..

[107] Wang X, Yang Y, Tan X, Mao X, Wei D, Yao Y (2019). Identification of tRNA-Derived Fragments Expression Profile in Breast Cancer Tissues. Curr Genomics.

[108] Zhou J, Wan F, Wang Y, Long J, Zhu X (2019). Small RNA sequencing reveals a novel tsRNA-26576 mediating tumorigenesis of breast cancer. Cancer Manag Res.

[109] Koi Y, Tsutani Y, Nishiyama Y, Ueda D, Ibuki Y, Sasada S (2020). Predicting the presence of breast cancer using circulating small RNAs, including those in the extracellular vesicles. Cancer Sci.

[110] Mo D, Jiang P, Yang Y, Mao X, Tan X, Tang X (2019). A tRNA fragment, 5'-tiRNA(Val), suppresses the Wnt/β-catenin signaling pathway by targeting FZD3 in breast cancer. Cancer Lett.

[111] Mo D, He F, Zheng J, Chen H, Tang L, Yan F (2021). tRNA-Derived Fragment tRF-17-79MP9PP Attenuates Cell Invasion and Migration via THBS1/TGF-β1/Smad3 Axis in Breast Cancer. Front Oncol.

[112] Zhang Y, Bi Z, Dong X, Yu M, Wang K, Song X (2021). tRNA-derived fragments: tRF-Gly-CCC-046, tRF-Tyr-GTA-010 and tRF-Pro-TGG-001 as novel diagnostic biomarkers for breast cancer. Thorac Cancer.

[113] Feng W, Li Y, Chu J, Li J, Zhang Y, Ding X (2018). Identification of tRNA-derived small noncoding RNAs as potential biomarkers for prediction of recurrence in triple-negative breast cancer. Cancer Med.

[114] Huang Y, Ge H, Zheng M, Cui Y, Fu Z, Wu X (2020). Serum tRNA-derived fragments (tRFs) as potential candidates for diagnosis of nontriple negative breast cancer. J Cell Physiol.

[115] Falconi M, Giangrossi M, Zabaleta ME, Wang J, Gambini V, Tilio M (2019). A novel 3'-tRNA(Glu)-derived fragment acts as a tumor suppressor in breast cancer by targeting nucleolin. Faseb j.

[116] Hussain SA, Deepak KV, Nanjappa DP, Sherigar V, Nandan N, Suresh PS (2021). Comparative expression analysis of tRF-3001a and tRF-1003 with corresponding miRNAs (miR-1260a and miR-4521) and their network analysis with breast cancer biomarkers. Mol Biol Rep.

[117] Tong L, Zhang W, Qu B, Zhang F, Wu Z, Shi J (2020). The tRNA-Derived Fragment-3017A Promotes Metastasis by Inhibiting NELL2 in Human Gastric Cancer. Front Oncol.

[118] Zhang F, Shi J, Wu Z, Gao P, Zhang W, Qu B (2020). A 3'-tRNA-derived fragment enhances cell proliferation, migration and invasion in gastric cancer by targeting FBXO47. Arch Biochem Biophys.

[119] Cui H, Li H, Wu H, Du F, Xie X, Zeng S (2022). A novel 3’tRNA-derived fragment tRF-Val promotes proliferation and inhibits apoptosis by targeting EEF1A1 in gastric cancer. Cell Death Dis.

[120] Gu X, Ma S, Liang B, Ju S (2021). Serum hsa_tsr016141 as a Kind of tRNA-Derived Fragments Is a Novel Biomarker in Gastric Cancer. Front Oncol.

[121] Zhang Y, Gu X, Qin X, Huang Y, Ju S (2022). Evaluation of serum tRF-23-Q99P9P9NDD as a potential biomarker for the clinical diagnosis of gastric cancer. Mol Med.

[122] Huang Y, Zhang H, Gu X, Qin S, Zheng M, Shi X (2021). Elucidating the Role of Serum tRF-31-U5YKFN8DYDZDD as a Novel Diagnostic Biomarker in Gastric Cancer (GC). J Oncol.

[123] Xu W, Zheng J, Wang X, Zhou B, Chen H, Li G (2022). tRF-Val-CAC-016 modulates the transduction of CACNA1d-mediated MAPK signaling pathways to suppress the proliferation of gastric carcinoma. Mol Med.

[124] Xu W, Zhou B, Wang J, Tang L, Hu Q, Wang J (2021). tRNA-Derived Fragment tRF-Glu-TTC-027 Regulates the Progression of Gastric Carcinoma via MAPK Signaling Pathway. Front Oncol.

[125] Dong X, Fan X, He X, Chen S, Huang W, Gao J (2020). Comprehensively Identifying the Key tRNA-Derived Fragments and Investigating Their Function in Gastric Cancer Processes. Onco Targets Ther.

[126] Wang H, Huang W, Fan X, He X, Chen S, Yu S (2022). The tRNA-Derived Fragment tRF-24-V29K9UV3IU Functions as a miRNA-like RNA to Prevent Gastric Cancer Progression by Inhibiting GPR78 Expression. J Oncol.

[127] Zheng J, Li C, Zhu Z, Yang F, Wang X, Jiang P (2022). A 5`-tRNA Derived Fragment Named tiRNA-Val-CAC-001 Works as a Suppressor in Gastric Cancer. Cancer Manag Res.

[128] Zhu L, Li T, Shen Y, Yu X, Xiao B, Guo J (2019). Using tRNA halves as novel biomarkers for the diagnosis of gastric cancer. Cancer Biomark.

[129] Shen Y, Xie Y, Yu X, Zhang S, Wen Q, Ye G (2021). Clinical diagnostic values of transfer RNA-derived fragment tRF-19-3L7L73JD and its effects on the growth of gastric cancer cells. J Cancer.

[130] Chen H, Xu Z, Cai H, Peng Y, Yang L, Wang Z (2022). Identifying Differentially Expressed tRNA-Derived Small Fragments as a Biomarker for the Progression and Metastasis of Colorectal Cancer. Dis Markers.

[131] Tsiakanikas P, Adamopoulos PG, Tsirba D, Artemaki PI, Papadopoulos IN, Kontos CK (2022). High Expression of a tRNAPro Derivative Associates with Poor Survival and Independently Predicts Colorectal Cancer Recurrence. Biomedicines.

[132] Wang X, Zhang Y, Ghareeb WM, Lin S, Lu X, Huang Y (2020). A Comprehensive Repertoire of Transfer RNA-Derived Fragments and Their Regulatory Networks in Colorectal Cancer. J Comput Biol.

[133] Zhu Y, Chen S, Ling Z, Winnicki A, Xu L, Xu S (2021). Comprehensive Analysis of a tRNA-Derived Small RNA in Colorectal Cancer. Front Oncol.

[134] Li S, Shi X, Chen M, Xu N, Sun D, Bai R (2019). Angiogenin promotes colorectal cancer metastasis via tiRNA production. Int J Cancer.

[135] Wu Y, Yang X, Jiang G, Zhang H, Ge L, Chen F (2021). 5'-tRF-GlyGCC: a tRNA-derived small RNA as a novel biomarker for colorectal cancer diagnosis. Genome Med.

[136] Huang B, Yang H, Cheng X, Wang D, Fu S, Shen W (2017). tRF/miR-1280 Suppresses Stem Cell-like Cells and Metastasis in Colorectal Cancer. Cancer Res.

[137] Hu F, Niu Y, Mao X, Cui J, Wu X, Simone CB (2021). tsRNA-5001a promotes proliferation of lung adenocarcinoma cells and is associated with postoperative recurrence in lung adenocarcinoma patients. Transl Lung Cancer Res.

[138] Shao Y, Sun Q, Liu X, Wang P, Wu R, Ma Z (2017). tRF-Leu-CAG promotes cell proliferation and cell cycle in non-small cell lung cancer. Chem Biol Drug Des.

[139] Yang W, Gao K, Qian Y, Huang Y, Xiang Q, Chen C (2022). A novel tRNA-derived fragment AS-tDR-007333 promotes the malignancy of NSCLC via the HSPB1/MED29 and ELK4/MED29 axes. J Hematol Oncol.

[140] Wang J, Liu X, Cui W, Xie Q, Peng W, Zhang H (2022). Plasma tRNA-derived small RNAs signature as a predictive and prognostic biomarker in lung adenocarcinoma. Cancer Cell Int.

[141] Li J, Cao C, Fang L, Yu W. Serum transfer RNA-derived fragment tRF-31–79MP9P9NH57SD acts as a novel diagnostic biomarker for non-small cell lung cancer. J Clin Lab Anal. 2022;36(7):e24492. 10.1002/jcla.24492 PMC9279995 35576497

[142] Gu W, Shi J, Liu H, Zhang X, Zhou JJ, Li M (2020). Peripheral blood non-canonical small non-coding RNAs as novel biomarkers in lung cancer. Mol Cancer.

[143] Fan H, Liu H, Lv Y, Song Y (2022). AS-tDR-007872: A Novel tRNA-Derived Small RNA Acts an Important Role in Non-Small-Cell Lung Cancer. Comput Math Methods Med.

[144] Karousi P, Adamopoulos PG, Papageorgiou SG, Pappa V, Scorilas A, Kontos CK (2020). A novel, mitochondrial, internal tRNA-derived RNA fragment possesses clinical utility as a molecular prognostic biomarker in chronic lymphocytic leukemia. Clin Biochem.

[145] Katsaraki K, Artemaki PI, Papageorgiou SG, Pappa V, Scorilas A, Kontos CK (2019). Identification of a novel, internal tRNA-derived RNA fragment as a new prognostic and screening biomarker in chronic lymphocytic leukemia, using an innovative quantitative real-time PCR assay. Leuk Res.

[146] Guo Y, Strickland SA, Mohan S, Li S, Bosompem A, Vickers KC (2017). MicroRNAs and tRNA-derived fragments predict the transformation of myelodysplastic syndromes to acute myeloid leukemia. Leuk Lymphoma.

[147] Katsaraki K, Adamopoulos PG, Papageorgiou SG, Pappa V, Scorilas A, Kontos CK (2021). A 3' tRNA-derived fragment produced by tRNA(LeuAAG) and tRNA(LeuTAG) is associated with poor prognosis in B-cell chronic lymphocytic leukemia, independently of classical prognostic factors. Eur J Haematol.

[148] Karousi P, Katsaraki K, Papageorgiou SG, Pappa V, Scorilas A, Kontos CK (2019). Identification of a novel tRNA-derived RNA fragment exhibiting high prognostic potential in chronic lymphocytic leukemia. Hematol Oncol.

[149] Jin L, Zhu C, Qin X (2019). Expression profile of tRNA-derived fragments in pancreatic cancer. Oncol Lett.

[150] Sui S, Wang Z, Cui X, Jin L, Zhu C (2022). The biological behavior of tRNA-derived fragment tRF-Leu-AAG in pancreatic cancer cells. Bioengineered.

[151] Jin F, Yang L, Wang W, Yuan N, Zhan S, Yang P (2021). A novel class of tsRNA signatures as biomarkers for diagnosis and prognosis of pancreatic cancer. Mol Cancer.

[152] Xue M, Shi M, Xie J, Zhang J, Jiang L, Deng X (2021). Serum tRNA-derived small RNAs as potential novel diagnostic biomarkers for pancreatic ductal adenocarcinoma. Am J Cancer Res.

[153] Li J, Jin L, Gao Y, Gao P, Ma L, Zhu B (2021). Low expression of tRF-Pro-CGG predicts poor prognosis in pancreatic ductal adenocarcinoma. J Clin Lab Anal.

[154] Pan L, Huang X, Liu ZX, Ye Y, Li R, Zhang J (2021). Inflammatory cytokine-regulated tRNA-derived fragment tRF-21 suppresses pancreatic ductal adenocarcinoma progression. J Clin Invest.

[155] Zhou Y, Hu J, Liu L, Yan M, Zhang Q, Song X (2021). Gly-tRF enhances LCSC-like properties and promotes HCC cells migration by targeting NDFIP2. Cancer Cell Int.

[156] Zhu L, Li J, Gong Y, Wu Q, Tan S, Sun D (2019). Exosomal tRNA-derived small RNA as a promising biomarker for cancer diagnosis. Mol Cancer.

[157] Peng EY, Shu Y, Wu Y, Zeng F, Tan S, Deng Y (2018). Presence and diagnostic value of circulating tsncRNA for ovarian tumor. Mol Cancer.

[158] Panoutsopoulou K, Dreyer T, Dorn J, Obermayr E, Mahner S, Gorp TV (2021). tRNA(GlyGCC)-Derived Internal Fragment (i-tRF-GlyGCC) in Ovarian Cancer Treatment Outcome and Progression. Cancers (Basel)..

[159] Zhang M, Li F, Wang J, He W, Li Y, Li H (2019). tRNA-derived fragment tRF-03357 promotes cell proliferation, migration and invasion in high-grade serous ovarian cancer. Onco Targets Ther.

[160] Qin C, Chen ZH, Cao R, Shi MJ, Tian Y (2022). Differential Expression Profiles and Bioinformatics Analysis of tRNA-Derived Small RNAs in Muscle-Invasive Bladder Cancer in a Chinese Population. Genes (Basel).

[161] Papadimitriou MA, Avgeris M, Levis P, Papasotiriou EC, Kotronopoulos G, Stravodimos K (2020). tRNA-Derived Fragments (tRFs) in Bladder Cancer: Increased 5'-tRF-LysCTT Results in Disease Early Progression and Patients' Poor Treatment Outcome. Cancers (Basel).

[162] Nientiedt M, Deng M, Schmidt D, Perner S, Müller SC, Ellinger J (2016). Identification of aberrant tRNA-halves expression patterns in clear cell renal cell carcinoma. Sci Rep.

[163] Zhao C, Tolkach Y, Schmidt D, Kristiansen G, Müller SC, Ellinger J (2018). 5'-tRNA Halves are Dysregulated in Clear Cell Renal Cell Carcinoma. J Urol.

[164] Li K, Lin Y, Luo Y, Xiong X, Wang L, Durante K (2022). A signature of saliva-derived exosomal small RNAs as predicting biomarker for esophageal carcinoma: a multicenter prospective study. Mol Cancer.

[165] Xu C, Fu Y (2021). Expression Profiles of tRNA-Derived Fragments and Their Potential Roles in Multiple Myeloma. Onco Targets Ther.

[166] Karousi P, Papanota AM, Artemaki PI, Liacos CI, Patseas D, Mavrianou-Koutsoukou N (2021). tRNA Derivatives in Multiple Myeloma: Investigation of the Potential Value of a tRNA-Derived Molecular Signature. Biomedicines.

[167] Shi Z, Qu X, Guo C, Zhang L, Peng C, Xie Z (2021). Identification of clinical trait-related small RNA biomarkers with weighted gene co-expression network analysis for personalized medicine in endocervical adenocarcinoma. Aging (Albany NY).

[168] Shan S, Wang Y, Zhu C (2021). A comprehensive expression profile of tRNA-derived fragments in papillary thyroid cancer. J Clin Lab Anal.

[169] Han L, Lai H, Yang Y, Hu J, Li Z, Ma B (2021). A 5'-tRNA halve, tiRNA-Gly promotes cell proliferation and migration via binding to RBM17 and inducing alternative splicing in papillary thyroid cancer. J Exp Clin Cancer Res.

[170] Maute RL, Schneider C, Sumazin P, Holmes A, Califano A, Basso K (2013). tRNA-derived microRNA modulates proliferation and the DNA damage response and is down-regulated in B cell lymphoma. Proc Natl Acad Sci U S A.

[171] Lu Z, Su K, Wang X, Zhang M, Ma S, Li H (2021). Expression Profiles of tRNA-Derived Small RNAs and Their Potential Roles in Primary Nasopharyngeal Carcinoma. Front Mol Biosci.

[172] Deng H, Wang J, Ye D, Chen J, Qiu S, Tang M (2022). A 5'-tiRNA fragment that inhibits proliferation and migration of laryngeal squamous cell carcinoma by targeting PIK3CD. Genomics.

[173] Gu X, Wang L, Coates PJ, Boldrup L, Fåhraeus R, Wilms T (2020). Transfer-RNA-Derived Fragments Are Potential Prognostic Factors in Patients with Squamous Cell Carcinoma of the Head and Neck. Genes (Basel).

[174] Dhahbi J, Nunez Lopez YO, Schneider A, Victoria B, Saccon T, Bharat K (2019). Profiling of tRNA Halves and YRNA Fragments in Serum and Tissue From Oral Squamous Cell Carcinoma Patients Identify Key Role of 5' tRNA-Val-CAC-2-1 Half. Front Oncol.

[175] Londin E, Magee R, Shields CL, Lally SE, Sato T, Rigoutsos I (2020). IsomiRs and tRNA-derived fragments are associated with metastasis and patient survival in uveal melanoma. Pigment Cell Melanoma Res.

[176] Xu B, Liang J, Zou H, Wang J, Xiong Y, Pei J (2022). Identification of Novel tRNA-Leu-CAA-Derived tsRNAs for the Diagnosis and Prognosis of Diffuse Gliomas. Cancer Manag Res.

[177] Li YK, Yan LR, Wang A, Jiang LY, Xu Q, Wang BG (2022). RNA-sequencing reveals the expression profiles of tsRNAs and their potential carcinogenic role in cholangiocarcinoma. J Clin Lab Anal..

[178] Guzman N, Agarwal K, Asthagiri D, Yu L, Saji M, Ringel MD (2015). Breast Cancer-Specific miR Signature Unique to Extracellular Vesicles Includes "microRNA-like" tRNA Fragments. Mol Cancer Res.

[179] Cui Y, Huang Y, Wu X, Zheng M, Xia Y, Fu Z (2019). Hypoxia-induced tRNA-derived fragments, novel regulatory factor for doxorubicin resistance in triple-negative breast cancer. J Cell Physiol.

[180] Zhu P, Lu J, Zhi X, Zhou Y, Wang X, Wang C (2021). tRNA-derived fragment tRFLys-CTT-010 promotes triple-negative breast cancer progression by regulating glucose metabolism via G6PC. Carcinogenesis.

[181] Farina NH, Scalia S, Adams CE, Hong D, Fritz AJ, Messier TL (2020). Identification of tRNA-derived small RNA (tsRNA) responsive to the tumor suppressor, RUNX1, in breast cancer. J Cell Physiol.

[182] Sung H, Ferlay J, Siegel RL, Laversanne M, Soerjomataram I, Jemal A (2021). Global Cancer Statistics 2020: GLOBOCAN Estimates of Incidence and Mortality Worldwide for 36 Cancers in 185 Countries. CA Cancer J Clin.

[183] Xiong W, Wang X, Cai X, Xiong W, Liu Y, Li C (2019). Identification of tRNA-derived fragments in colon cancer by comprehensive small RNA sequencing. Oncol Rep.

[184] Luan N, Chen Y, Li Q, Mu Y, Zhou Q, Ye X (2021). TRF-20-M0NK5Y93 suppresses the metastasis of colon cancer cells by impairing the epithelial-to-mesenchymal transition through targeting Claudin-1. Am J Transl Res.

[185] Cao KY, Pan Y, Yan TM, Tao P, Xiao Y, Jiang ZH (2022). Antitumor Activities of tRNA-Derived Fragments and tRNA Halves from Non-pathogenic Escherichia coli Strains on Colorectal Cancer and Their Structure-Activity Relationship. mSystems..

[186] Umu SU, Langseth H, Zuber V, Helland Å, Lyle R, Rounge TB (2022). Serum RNAs can predict lung cancer up to 10 years prior to diagnosis. Elife.

[187] Zhan S, Yang P, Zhou S, Xu Y, Xu R, Liang G (2022). Serum mitochondrial tsRNA serves as a novel biomarker for hepatocarcinoma diagnosis. Front Med.

[188] Zuo Y, Chen S, Yan L, Hu L, Bowler S, Zitello E (2022). Development of a tRNA-derived small RNA diagnostic and prognostic signature in liver cancer. Genes Dis.

[189] Chen B, Liu S, Wang H, Li G, Lu X, Xu H (2021). Differential Expression Profiles and Function Prediction of Transfer RNA-Derived Fragments in High-Grade Serous Ovarian Cancer. Biomed Res Int.

[190] Zhou K, Diebel KW, Holy J, Skildum A, Odean E, Hicks DA (2017). A tRNA fragment, tRF5-Glu, regulates BCAR3 expression and proliferation in ovarian cancer cells. Oncotarget.

[191] Cao KY, Yan TM, Zhang JZ, Chan TF, Li J, Li C (2022). A tRNA-derived fragment from Chinese yew suppresses ovarian cancer growth via targeting TRPA1. Mol Ther Nucleic Acids.

[192] Yang C, Lee M, Song G, Lim W (2021). tRNA(Lys)-Derived Fragment Alleviates Cisplatin-Induced Apoptosis in Prostate Cancer Cells. Pharmaceutics..

[193] Su Z, Monshaugen I, Wilson B, Wang F, Klungland A, Ougland R (2022). TRMT6/61A-dependent base methylation of tRNA-derived fragments regulates gene-silencing activity and the unfolded protein response in bladder cancer. Nat Commun.

[194] Kazimierczyk M, Wojnicka M, Biała E, Żydowicz-Machtel P, Imiołczyk B, Ostrowski T (2022). Characteristics of Transfer RNA-Derived Fragments Expressed during Human Renal Cell Development: The Role of Dicer in tRF Biogenesis. Int J Mol Sci.

[195] Shi J, Zhang Y, Tan D, Zhang X, Yan M, Zhang Y (2021). PANDORA-seq expands the repertoire of regulatory small RNAs by overcoming RNA modifications. Nat Cell Biol.

[196] Victoria Martinez B, Dhahbi JM, Nunez Lopez YO, Lamperska K, Golusinski P, Luczewski L (2015). Circulating small non-coding RNA signature in head and neck squamous cell carcinoma. Oncotarget.

[197] Ma J, Liu F (2022). Study of tRNA-Derived Fragment tRF-20-S998LO9D in Pan-Cancer. Dis Markers.

[198] Tosar JP, Gámbaro F, Sanguinetti J, Bonilla B, Witwer KW, Cayota A (2015). Assessment of small RNA sorting into different extracellular fractions revealed by high-throughput sequencing of breast cell lines. Nucleic Acids Res.

[199] Ren J, Zhou Q, Li H, Li J, Pang L, Su L, et al. Characterization of exosomal RNAs derived from human gastric cancer cells by deep sequencing. Tumour Biol. 2017;39(4):1010428317695012. 10.1177/1010428317695012 28381156

[200] Tong F, Andress A, Tang G, Liu P, Wang X (2020). Comprehensive profiling of extracellular RNA in HPV-induced cancers using an improved pipeline for small RNA-seq analysis. Sci Rep.

[201] Wei Z, Batagov AO, Schinelli S, Wang J, Wang Y, El Fatimy R (2017). Coding and noncoding landscape of extracellular RNA released by human glioma stem cells. Nat Commun.

